# Far-Field Non-Contact Eye Tracking for Visual Attention Analysis in Runway Viewing Environments

**DOI:** 10.3390/jemr19050117

**Published:** 2026-10-08

**Authors:** Hannuo Tian, Ding-Bang Luh, Mingyang Guo, Qianli Lin, Jun Ma

**Affiliations:** 1 School of Art and Design, Guangdong University of Technology, Guangzhou 510090, China; tianhannuopro@163.com (H.T.); luhdingbang@126.com (D.-B.L.); 2 College of Automotive Studies, Tongji University, Shanghai 201804, China; gmingyang1998@163.com; 3 College of Design and Innovation, Tongji University, Shanghai 200092, China; luliqianli@gmail.com

**Keywords:** non-contact eye tracking, runway viewing, remote gaze estimation, visual attention, stacked hourglass network, homography mapping

## Abstract

Conventional eye trackers are difficult to deploy in fashion-runway settings because they require head stabilization, wearable devices, close-range cameras, or infrared illumination. This study develops a far-field, non-contact eye-tracking framework and prototype system for measuring target-directed visual attention in runway-viewing settings using standard cameras. The framework integrates eye-region normalization, 3D gaze estimation, individualized homography-based point-of-regard mapping onto a scene plane, adaptive filtering, DBSCAN-based outlier removal, and kernel density estimation. In Experiment 1, 16 participants completed a controlled calibration-grid benchmark with the proposed system and EyeLink 1000 Plus. Compared with the EyeLink 1000 Plus, the proposed system exhibited higher positional error ($A_{\mathrm{pos}}$: 2.50±0.25° vs. 1.74±0.55°) and a higher value on the processed-output spatial-precision measure ($A_{\mathrm{spa}}$: 2.31±0.24° vs. 1.02±0.37°). These results characterize end-to-end localization under the calibrated benchmark geometry and provide a controlled reference for the subsequent AOI-level evaluation. In Experiment 2, an independent sample of 16 participants completed an instructed right chest targeting task at 2 m, 4 m and 6 m. AOI-retained sample percentages were 80.4%, 97.3%, and 96.0%, respectively, and gaze-density maps concentrated within the predefined upper-body target region. These findings support the feasibility of target-directed AOI-level localization at far-field distances without chin-rest stabilization under the tested simulated runway-observation protocol.

## 1. Introduction

Fashion shows are among the most concentrated forms of visual communication in contemporary creative industries [1]. Through meticulously orchestrated set designs, model performances, and spatial configurations, these events serve as platforms for conveying brand identity, cultural values, and evolving aesthetic sensibilities [2]. Key Opinion Leaders (KOLs) positioned in runway observation areas play a central role in aesthetic evaluation and content dissemination. Their patterns of visual attention are therefore commercially and culturally relevant to understanding fashion-show information delivery and garment presentation [3,4]. Examining how KOLs allocate attention across garment and body regions during runway performances is consequently of practical significance.

Conventional approaches to studying KOL preferences have relied primarily on post-hoc subjective methods such as questionnaires and semi-structured interviews [3,5], which are fundamentally limited in their capacity to reconstruct the fine-grained, moment-by-moment visual selection process during live viewing. Eye-tracking offers a more direct approach by recording time-resolved gaze behavior, enabling objective characterization of overt visual attention and information-sampling patterns [6,7,8]. Although eye-tracking has been applied across human–computer interaction, marketing, and fashion retail contexts to quantify attentional allocation [9,10,11], these studies have predominantly relied on screen-based or simulated environments whereas direct empirical investigation in live runway-observation settings remains limited.

In parallel, appearance-based gaze estimation using deep learning has attracted considerable research attention as a flexible alternative to hardware-intensive approaches [12,13]. Unlike corneal-reflection systems that depend on active infrared illumination [14], appearance-based gaze-estimation methods infer gaze from facial or ocular appearance captured by conventional cameras, reducing reliance on specialized active-infrared hardware [12,13]. Recent advances have addressed a range of challenges including semi-supervised landmark detection [15], iris geometry modeling [16], cross-subject appearance debiasing [17], cross-domain generalization [18], and calibration-free webcam-based estimation [19]. Park et al. [20] demonstrated that structurally interpretable ocular keypoints estimated from ordinary images provide a robust basis for model-based gaze estimation. At the same time, previous evaluations of remote eye trackers have shown that data quality can vary with head position and acquisition geometry [21], while recent reviews have identified substantial heterogeneity in working distance, measurement procedures, and reported oculomotor parameters across studies [22]. These findings indicate that gaze-estimation performance should be interpreted with respect to the specific recording geometry and application context in which it is measured.

Despite this collective progress, applying gaze estimation to visual attention analysis in live runway scenarios remains substantially underexplored. First, existing studies of KOL and influencer preferences frequently rely on retrospective self-report methods such as surveys and interviews [3,5], which cannot capture time-resolved visual selection during live viewing; second, existing eye-tracking technologies involve trade-offs among measurement accuracy, working distance, participant mobility, hardware requirements, and deployment complexity, which can make their use difficult in runway observation settings [23,24,25]; third, much of the appearance-based gaze-estimation literature focuses on gaze-direction or gaze-point estimation and generalization, whereas application-specific mapping of these estimates to interpretable scene-level AOIs remains a separate methodological step [12]; fourth, runway observation requires incorporating changes in the distance between the observer and the target, and more importantly, it requires interpreting gaze estimation based on clothing or body regions within the scene space.

Motivated by these limitations, this paper proposes a far-field non-contact eye-tracking framework for target-directed visual-attention measurement in runway-viewing environments. The framework integrates four core modules: (1) camera-based eye-image acquisition and preprocessing; (2) 3D gaze estimation via a Park et al. [20]-based stacked hourglass network with multi-stage heatmap refinement; (3) gaze-to-point-of-regard (POR) mapping through a homography scene coordinate transformation; and (4) attention analysis and visualization using adaptive filtering, density-based clustering, and kernel density estimation (KDE). A prototype system implementing this framework was developed and evaluated through two experiments: a controlled post-calibration benchmark comparison against the EyeLink 1000 Plus (SR Research Ltd., Ottawa, Canada), and an applied experiment in a simulated runway observation scenario. The principal contributions are as follows.

(1) We propose a far-field, non-contact eye-tracking framework that combines appearance-based 3D gaze-direction estimation with individualized homography-based planar gaze-to-scene mapping for scene-level AOI analysis using standard cameras.
(2) We design a dedicated attention-analysis pipeline that combines adaptive One Euro filtering, Density-based spatial clustering of applications with noise (DBSCAN)-based outlier removal, and KDE to convert noisy POR sequences into stable, interpretable area-of-interest (AOI)-level gaze representations suitable for garment- and design-level analysis.
(3) We implement the framework as a prototype system with real-time visualization, parameter configuration, and structured data export, supporting operation by researchers without specialized programming expertise.
(4) We provide an application-specific empirical evaluation of the framework: a controlled post-calibration benchmark against a commercial infrared eye tracker at predefined calibration-grid locations, and a target-directed runway-observation experiment with an independent participant sample at viewing distances of 2 m, 4 m and 6 m. These experiments separately characterize controlled positional performance and application-oriented AOI-level localization.

## 2. Materials and Methods

### 2.1. Overall Framework

**Figure 1:**
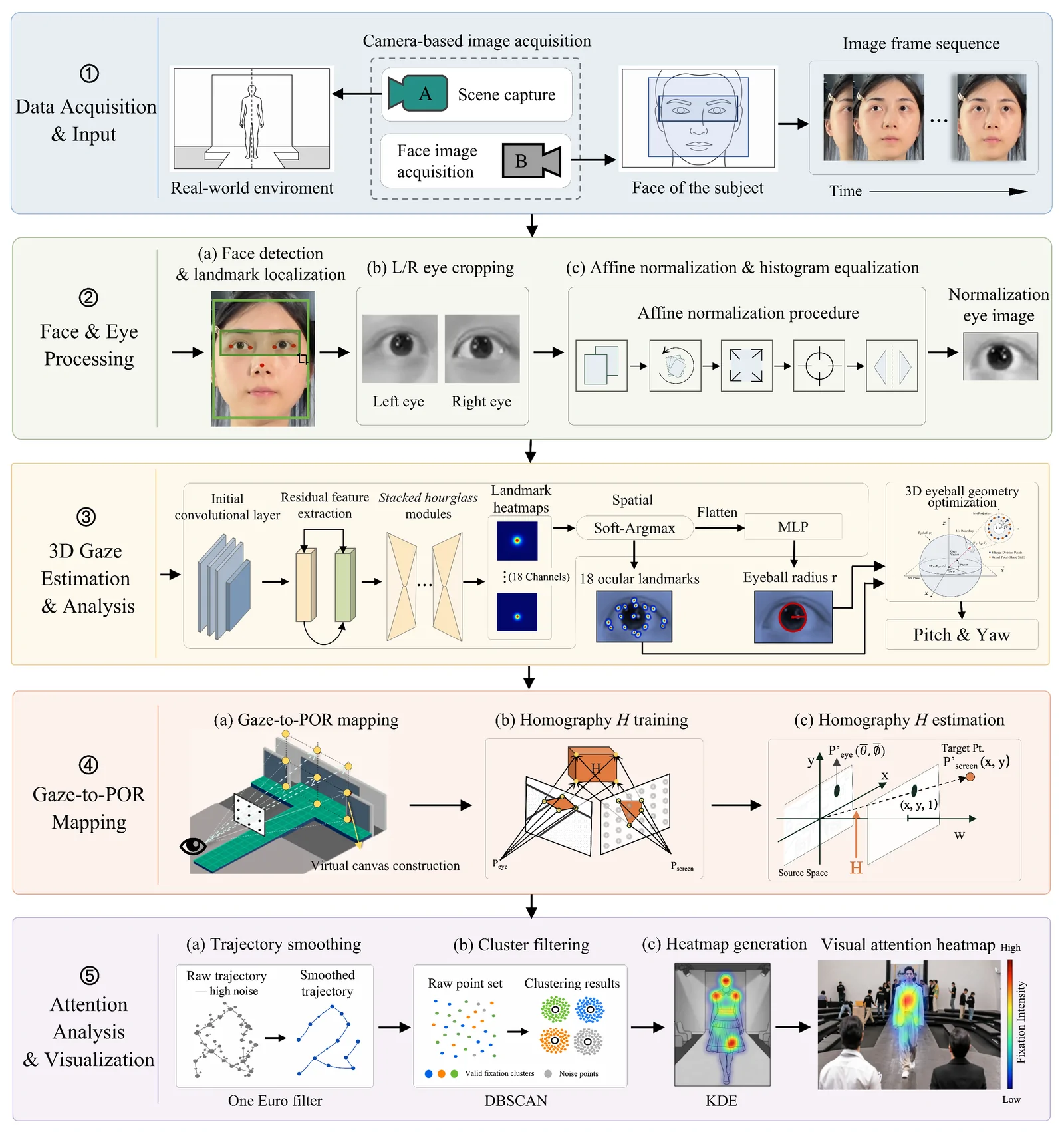
Overall framework of the proposed far-field non-contact eye-tracking system. Note. Arrows between processing blocks indicate the workflow. A and B denote scene-image and face-image acquisition, respectively, and *H* denotes the homography matrix. Colored backgrounds distinguish the processing stages, whose headings correspond to the relevant methodological subsections.

Figure 1 illustrates the overall architecture of the proposed framework, which consists of four main functional modules. The Data Acquisition and Eye-Region Preprocessing module acquires facial and ocular signals from standard RGB camera video and converts each frame into normalized eye-region images. The 3D Gaze Estimation and Analysis module estimates ocular structural landmarks using a stacked hourglass network and calculates gaze direction from a 3D eyeball geometry model. The Gaze-to-POR Mapping module converts estimated gaze directions into scene-level points of regard through an individualized calibration procedure and homography transformation. The Attention Analysis and Visualization module generates AOI-based attention representations for subsequent visual behavior analysis.

### 2.2. Data Acquisition and Eye-Region Processing

#### 2.2.1. Face and Eye Region Detection

Face region detection is the foundation of eye-tracking analysis. The camera acquires input image frames, which are mirrored and converted to grayscale. Face detection employs the maximum-margin object detection (MMOD)-based convolutional neural network detector provided by the dlib library [26,27], offering robust localization under varying poses and lighting conditions. To reduce computational overhead, input images are downsampled to half resolution prior to inference, with resulting bounding boxes scaled back by a factor of 2. Following face detection, dlib’s five-point shape predictor was used to localize the inner and outer corners of both eyes together with a nose landmark [26,28], providing the landmarks used for eye-region extraction.

#### 2.2.2. Landmark-Based 2D Affine Alignment of the Eye Region

To reduce image-plane variation in eye position, in-plane rotation, and scale during viewing, a composite 2D affine transformation was constructed for each eye to align the detected eye region to a standardized input image of fixed size (36×60px in this work). The transformation consists of translation, in-plane rotation, isotropic scaling, and recentering. This preprocessing standardizes the local eye crop in the image plane and does not perform virtual-camera normalization or explicitly compensate for general 3D head-to-camera pose variation [13].

Translation $T_{i}$: the coordinate origin is shifted to the geometric center of the eye’s inner and outer corners:

(1) $$T_{i}=\left[\begin{array}{ccc} 1 & 0 & -c_{x}\\ 0 & 1 & -c_{y}\\ 0 & 0 & 1 \end{array}\right],$$

where $\left(c_{x},c_{y}\right)$ denotes the geometric center of the two eye corners.

Rotation $T_{r}$: let the inner and outer corner coordinates of either eye be $P_{l}\left(x_{l},y_{l}\right)$ and $P_{r}\left(x_{r},y_{r}\right)$ respectively. The tilt angle θ between the inter-corner axis and the image horizontal is:

(2) $$\theta =\arctan\left(\frac{y_{r}-y_{l}}{x_{r}-x_{l}}\right).$$

A reverse rotation of −θ corrects this tilt:

(3) $$T_{r}=\left[\begin{array}{ccc} \cos(-\theta ) & -\sin(-\theta ) & 0\\ \sin(-\theta ) & \cos(-\theta ) & 0\\ 0 & 0 & 1 \end{array}\right].$$

Scaling $T_{s}$: the current eye width is defined as $w_{e}=1.5\sqrt{{\left(x_{r}-x_{l}\right)}^{2}+{\left(y_{r}-y_{l}\right)}^{2}}$, where 1.5 preserves a contextual margin. The resulting eye width is then normalized to the preset output width $W_{0}=60\mathrm{px}$:

(4) $$s=\frac{W_{0}}{w_{e}},\;T_{s}=\left[\begin{array}{ccc} s & 0 & 0\\ 0 & s & 0\\ 0 & 0 & 1 \end{array}\right].$$

Recentering $T_{c}$: the scaled coordinate origin is translated to the center of the output image $\left(\frac{W_{0}}{2},\frac{H_{0}}{2}\right)$ where $W_{0}=60\mathrm{px}$ and $H_{0}=36\mathrm{px}$:

(5) $$T_{c}=\left[\begin{array}{ccc} 1 & 0 & W_{0}/2\\ 0 & 1 & H_{0}/2\\ 0 & 0 & 1 \end{array}\right].$$

The composite transformation $T=T_{c}\;\cdot \;T_{s}\;\cdot \;T_{r}\;\cdot \;T_{i}$ resamples the local eye region into a standardized image with uniform orientation and fixed resolution. To reduce distributional discrepancy from left–right mirror symmetry, the left-eye crop is horizontally flipped after extraction [29], enabling a single set of network weights to perform binocular inference. After normalization, Contrast-Limited Adaptive Histogram Equalization was applied to the cropped grayscale eye image to suppress local brightness inhomogeneity while avoiding overamplification of noise in near-uniform regions, followed by intensity normalization to the range [0,1] [30]. These operations reduce the influence of illumination variation on local texture distribution and improve the recognizability of key structures such as iris edges, eye corners, and eyelid contours [31]. The resulting image provides a reliable, standardized input for subsequent hourglass-based structural reconstruction.

### 2.3. 3D Gaze Estimation and Analysis

#### 2.3.1. Feature Extraction of Eye Images

**Figure 2:**
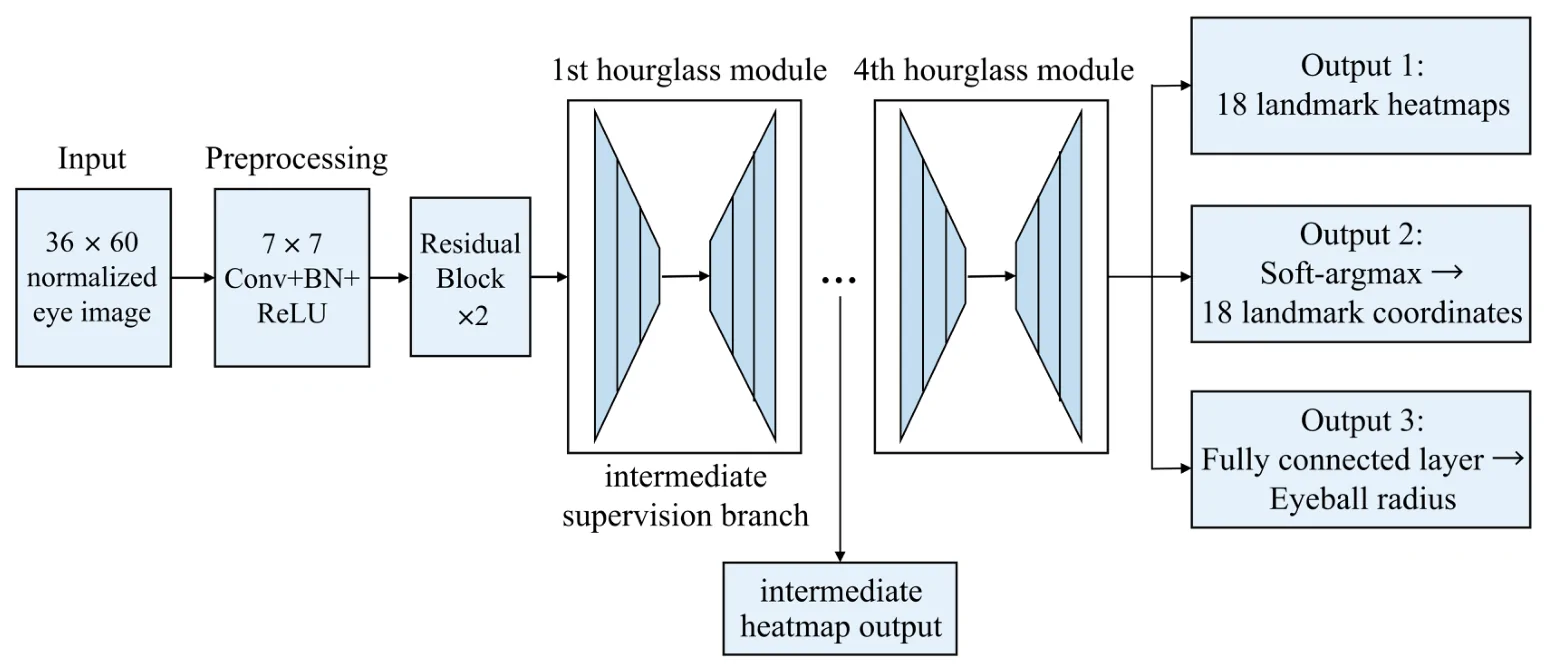
Stacked hourglass network architecture.

The trained network [20] was obtained using synthetic UnityEyes eye images and annotations, with extensive data augmentation and curriculum learning. Their reference model was optimized using Adam with a learning rate of $5\times 10^{-4}$, a batch size of 16, and was trained for 6.8 million steps. In the present study, the pretrained network weights were retained without parameter-level retraining, fine-tuning, or domain adaptation using participant images or runway recordings. As shown in Figure 2, the implementation comprises an initial convolutional stage, residual-block feature extraction, and a multi-stage stacked-hourglass module for localization of 18 ocular landmarks.

(1) Initial Convolutional Layer Setup

In the initial stage, the network performs preliminary feature extraction and resolution adjustment through a pre-convolutional module. Specifically: a 7×7 convolutional layer with stride $s_{0}=1$ and output channel count n=32 extracts low-level edge and texture features with a large receptive field; batch normalization (BN) and a rectified linear unit (ReLU) activation are then applied; finally, two residual blocks sequentially expand the feature-map channel count from *n* to 2n and then compress it back to *n*, while limiting feature-channel width and computational complexity. With $s_{0}=1$, the initial stage preserves the spatial resolution of the normalized eye crop, yielding an output feature map of H×W (H=36px, W=60px).

(2) Residual Block Design

Each residual block follows a bottleneck structure: a 1×1 convolution compresses channels from $n_{\mathrm{in}}$ to $\frac{n_{\mathrm{out}}}{2}$; a 3×3 convolution performs spatial feature fusion; a final 1×1 convolution restores channels to $n_{\mathrm{out}}$. BN and ReLU precede each convolution. Skip connections add the input directly when $n_{\mathrm{in}}=n_{\mathrm{out}}$, or via a 1×1 projection convolution otherwise, preventing gradient vanishing and ensuring trainability of the deep network.

(3) Hourglass Module Design

In the present configuration, each hourglass module is constructed through recursive nesting with depth $D_{\max}=4$, enabling feature integration across multiple spatial scales. At each recursion level *d*, the input feature map $x^{\left(d\right)}$ is divided into two parallel branches. The uplink path preserves spatial resolution and extracts fine local features through a residual block:

(6) $$u^{\left(d\right)}=R_{\mathrm{up}}^{\left(d\right)}\left(x^{\left(d\right)}\right),$$

where $R_{\mathrm{up}}^{\left(d\right)}$ denotes the independent residual block on the uplink path. The features retained here are rich in fine-grained edge-contour and local-texture information, forming the basis for precise localization. The downlink path first halves spatial resolution via max-pooling, then extracts global semantic features through a residual block yielding $L_{1}^{\left(d\right)}$. If $d<D_{\max}$, $L_{1}^{\left(d\right)}$ serves as input to the next recursive level; at the recursion bottom ($d=D_{\max}$):

(7) $$L_{2}^{\left(D_{\max}\right)}=R_{\mathrm{low}}^{\left(D_{\max}\right)}\left(L_{1}^{\left(D_{\max}\right)}\right),$$

where $R_{\mathrm{low}}^{\left(d\right)}$ denotes the independent residual block on the downlink path. After each level obtains $L_{2}^{\left(d\right)}$, a further residual block yields $L_{3}^{\left(d\right)}$, which is upsampled via bilinear interpolation to match $u^{\left(d\right)}$; element-wise summation fuses high-resolution local features with low-resolution global semantics, preserving a constant channel count throughout.

The landmark-learning formulation uses intermediate supervision after each of the first three hourglass modules, following standard stacked-hourglass practice [32]. During model training, a 1×1 convolution projects each module output onto the heatmap space, with mean squared error (MSE) used as the landmark-heatmap objective:

(8) $$L_{\mathrm{inter}}=\sum_{k=1}^{3}\sum_{j=1}^{18}{∥{\hat{H}}_{k,j}-H_{j}^{*}∥}_{F}^{2},$$

where $H_{j}^{*}$ is the ground-truth Gaussian heatmap for the *j*-th keypoint and ${\hat{H}}_{k,j}$ is the corresponding intermediate prediction from the *k*-th module. The final module’s output is supervised by the same loss, enhancing gradient flow to early layers and accelerating convergence. The final heatmap tensor has dimension $N\times 18\times H^{\prime }\times W^{\prime }$, representing 8 inner-eyelid boundary points, 8 iris-edge points, 1 iris center, and 1 eyeball center.

In the present implementation, the available stage-wise landmark heatmaps are averaged before Soft-Argmax coordinate extraction. This inference-time aggregation was adopted to combine the multiple supervised stage-wise predictions already produced by the stacked-hourglass network, rather than relying on a single stage output when processing the low-resolution eye crops used in the present system. The operation introduces no additional trainable parameters and does not modify the inherited pretrained weights. Because no ablation comparison was performed, this procedure is treated as an implementation design choice rather than as a demonstrated performance improvement.

(4) Keypoint Coordinate Extraction

Accurate 2D keypoint coordinates are extracted using the differentiable soft-Argmax operation, circumventing the resolution ceiling of hard-Argmax. A normalized coordinate grid matching the heatmap spatial size is pre-established, with horizontal grid ${\left\{x_{j}\right\}}_{j=0}^{W^{\prime }-1}$ and vertical grid ${\left\{y_{i}\right\}}_{i=0}^{H^{\prime }-1}$ expanded to matrices X and Y via meshgrid. For the flattened heatmap vector $H_{k}\in R^{H^{\prime }W^{\prime }}$, a temperature-scaled Softmax yields the normalized spatial probability distribution:

(9) $$p_{m}^{\left(k\right)}=\frac{\exp\left(\beta H_{k,m}\right)}{\sum_{m^{\prime }=1}^{H^{\prime }W^{\prime }}\exp\left(\beta H_{k,m^{\prime }}\right)},\;m=1,\ldots ,H^{\prime }W^{\prime }.$$

The temperature parameter β=100 sharpens the distribution toward the maximum-response location while preserving differentiability. The expected normalized coordinates are:

(10) $$\left\{\begin{array}{c} {\hat{x}}_{k}=\sum_{m=1}^{H^{\prime }W^{\prime }}p_{m}^{\left(k\right)}\;\cdot \;x_{\mathrm{ref},m},\\ {\hat{y}}_{k}=\sum_{m=1}^{H^{\prime }W^{\prime }}p_{m}^{\left(k\right)}\;\cdot \;y_{\mathrm{ref},m}. \end{array}\right.$$

The coordinates are mapped back to the pixel domain with a sub-pixel center offset:

(11) $$\left\{\begin{array}{c} x_{k}={\hat{x}}_{k}\left(W^{\prime }-1\right)+0.5,\\ y_{k}={\hat{y}}_{k}\left(H^{\prime }-1\right)+0.5. \end{array}\right.$$

This process is repeated for all 18 keypoints, yielding a coordinate tensor of shape N×18×2. Since the heatmap resolution is $\frac{1}{s}$ of the input image resolution, all coordinates are multiplied by the stride factor *s* to restore the original eye-image coordinate system.

(5) Eye Radius Regression

All 18 keypoint coordinates are flattened into a 36-dimensional structure vector and passed through a three-layer multilayer perceptron (MLP) (100 neurons per hidden layer, BN, ReLU), outputting a scalar eyeball radius estimate $\hat{r}$ through a final single-neuron fully connected layer. The eye-radius regression head uses mean absolute error (MAE) as its training objective, $L_{r}=\left|\hat{r}-r^{*}\right|$, where $r^{*}$ denotes the ground-truth radius label.

#### 2.3.2. Gaze Direction Calculation Based on the 3D Eyeball Model

**Figure 3:**
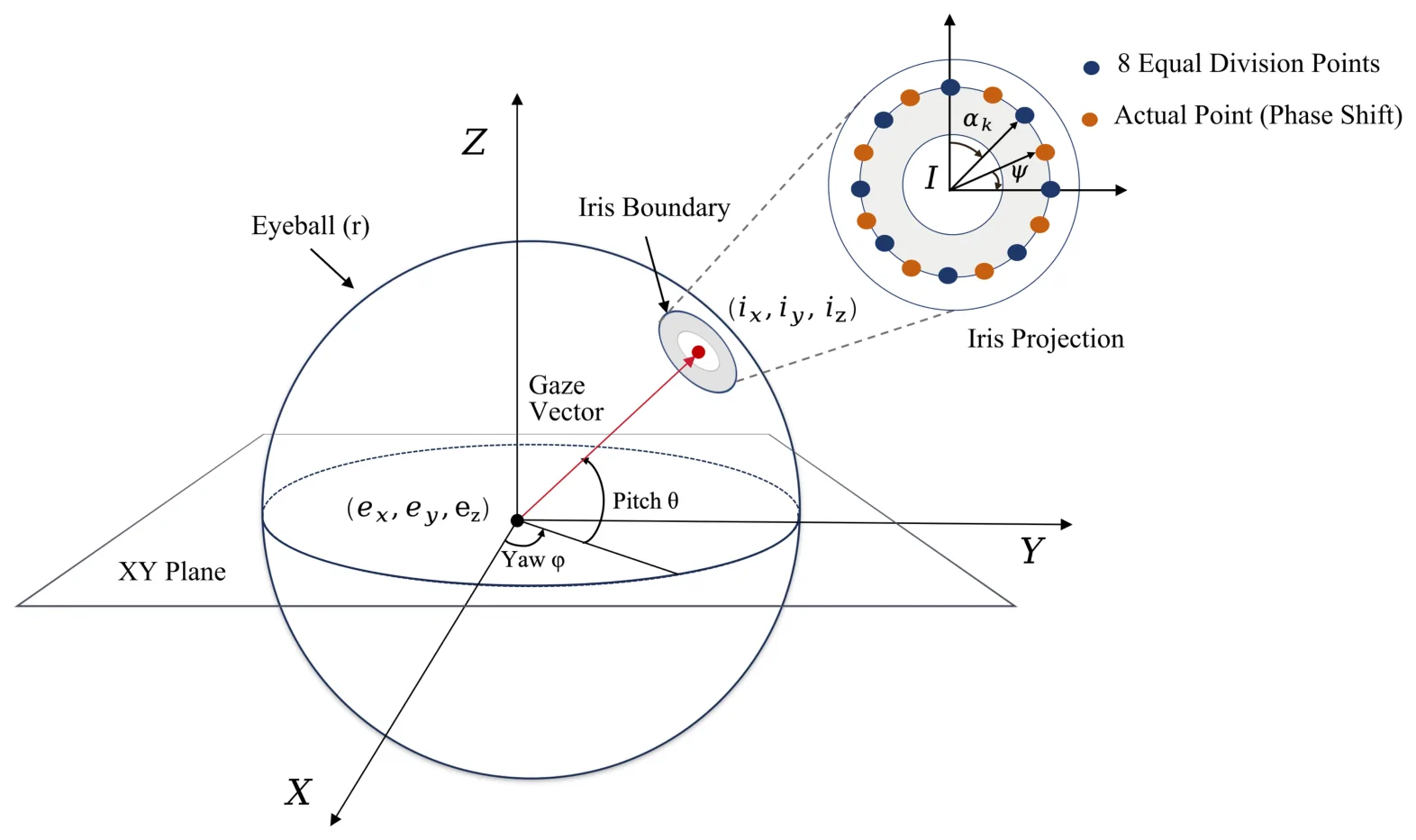
3D eyeball projection model. Note. The red dot marks the iris center $\left(i_{x},i_{y},i_{z}\right)$. The red line connects the eyeball center $\left(e_{x},e_{y},e_{z}\right)$ to the iris center and represents the gaze direction in the geometric model.

After obtaining the 18 keypoint coordinates and estimated eyeball radius $\hat{r}$, 3D gaze direction is estimated through geometric optimization of a 3D eyeball model [16,20], as shown in Figure 3. Let $\left(e_{x},e_{y},e_{z}\right)$ be the eyeball center coordinates, $\left(i_{x},i_{y},i_{z}\right)$ the iris center coordinates, and *r* the estimated radius. Gaze direction is parameterized by pitch angle θ (vertical deflection, positive upward) and yaw angle ϕ (horizontal deflection).

The 2D displacement of the iris center relative to the eyeball center, $\Delta x=i_{x}-e_{x}$ and $\Delta y=i_{y}-e_{y}$, yields closed-form initial estimates:

(12) $$\left\{\begin{array}{c} \theta_{0}=\arcsin\left(\frac{\Delta y}{r}\right),\\ \phi_{0}=\arcsin\left(\frac{-\Delta x}{r\cos\theta_{0}}\right). \end{array}\right.$$

These initial values provide a physically grounded starting point for the subsequent nonlinear optimization and substantially reduce the number of iterations required for convergence.

Relying solely on the iris-center displacement ignores the full 3D shape of the iris, limiting accuracy. Therefore, a nonlinear optimization step [16,20] is employed that incorporates all eight equally spaced iris-edge keypoints (azimuth angles $\alpha_{k}=\frac{k\pi }{4},\;k=0,\ldots ,7$) as geometric constraints. The parameter vector (θ,ϕ,δ,ψ) is jointly optimized, where δ is the angular displacement of the iris relative to the eyeball, and ψ is the initial phase shift of the iris circumference.

(13) $${\tilde{\alpha }}_{k}=\alpha_{k}+\psi ,\;\alpha_{k}=\frac{k\pi }{4},\;k=0,\ldots ,7.$$

The image-plane predicted coordinates of the *k*-th iris-edge point follow the 3D rotational projection relationship:

(14) $$\left\{\begin{array}{c} {\hat{x}}_{k}=e_{x}-r\cos\left(\theta +\delta \sin{\tilde{\alpha }}_{k}\right)\;\cdot \;\sin\left(\phi +\delta \cos{\tilde{\alpha }}_{k}\right),\\ {\hat{y}}_{k}=e_{y}+r\sin\left(\theta +\delta \sin{\tilde{\alpha }}_{k}\right). \end{array}\right.$$

The predicted iris-center pixel coordinates at the current gaze angle are:

(15) $$\left\{\begin{array}{c} {\hat{i}}_{x}=e_{x}+r\left(-\cos\theta \;\cdot \;\sin\phi \right),\\ {\hat{i}}_{y}=e_{y}+r\sin\theta . \end{array}\right.$$

The iris-center reprojection error is thus:

(16) $$\left\{\begin{array}{c} \Delta x_{c}={\hat{i}}_{x}-i_{x}=e_{x}-r\cos\theta \sin\phi -i_{x},\\ \Delta y_{c}={\hat{i}}_{y}-i_{y}=e_{y}+r\sin\theta -i_{y}. \end{array}\right.$$

The composite objective function minimizes both iris-edge and iris-center reprojection errors:

(17) $$L\left(\theta ,\phi ,\delta ,\psi \right)=\frac{1}{8}\sum_{k=0}^{7}\left[{\left({\hat{x}}_{k}-i_{x}^{\left(k\right)}\right)}^{2}+{\left({\hat{y}}_{k}-i_{y}^{\left(k\right)}\right)}^{2}\right]+\Delta x_{c}^{2}+\Delta y_{c}^{2},$$

where $\left(i_{x}^{\left(k\right)},i_{y}^{\left(k\right)}\right)$ are the network detection coordinates of the *k*-th iris edge keypoint. The optimization process calls the truncated Newton method with physical constraint boundaries for each parameter: θ∈[−0.4π,0.4π], ϕ∈[−0.4π,0.4π], δ∈[−0.01π,0.5π], and ψ∈[−π,π].

After convergence, the final estimated gaze angles (−θ,ϕ) are output. The sign negation on θ adapts to the image coordinate convention in which the y-axis points downward, ensuring that the output θ is positive for upward gaze.

For benchmark context, Park et al. [20] reported mean angular gaze errors of 7.1° on Columbia Gaze and 11.9° on EYEDIAP using their model-based gaze-estimation approach with 20 calibration images per participant.

### 2.4. Gaze-to-POR Mapping

**Figure 4:**
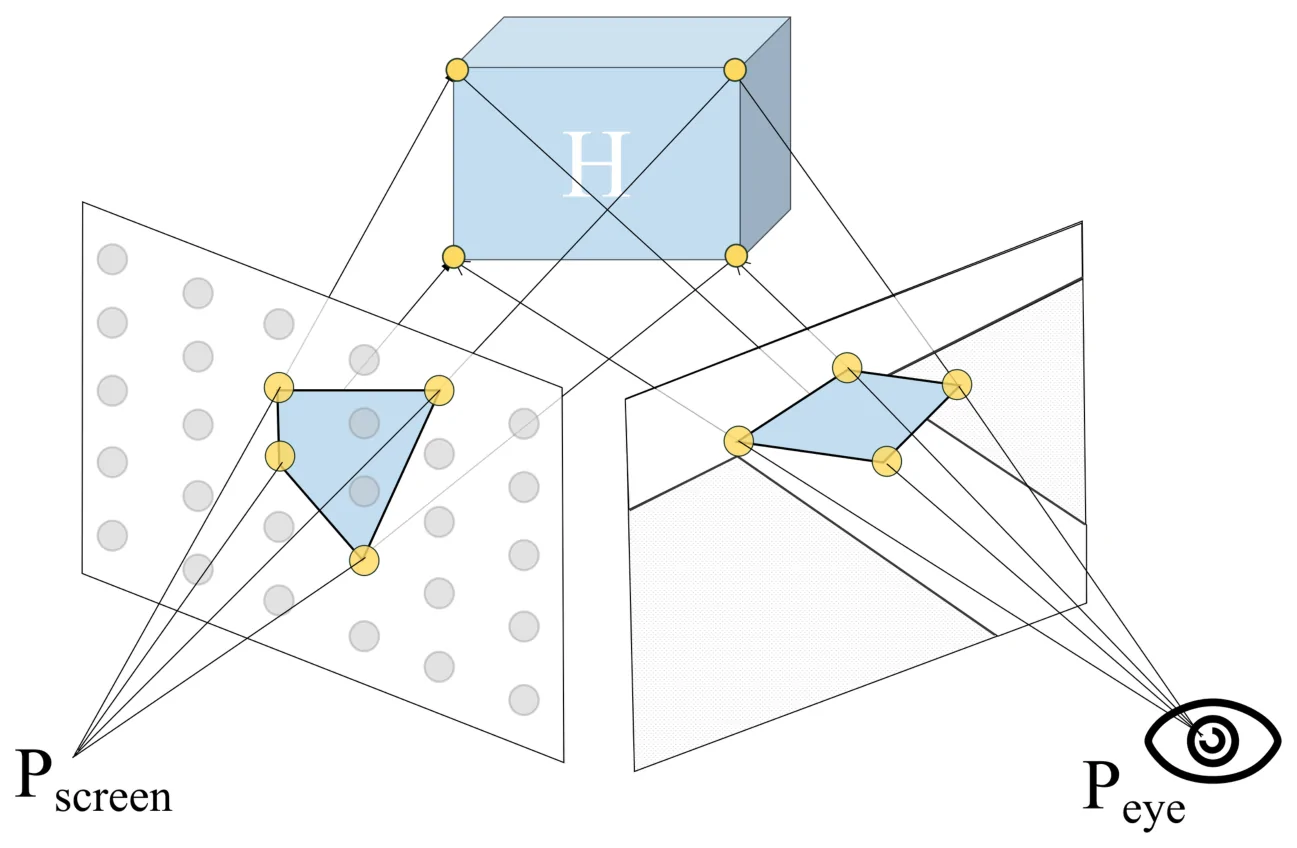
Schematic diagram of POR mapping based on homography transformation. Note. Yellow dots indicate corresponding points in the schematic. *H* denotes the homography matrix mapping normalized 2D gaze coordinates to scene-plane point-of-regard (POR) coordinates.

3D gaze estimation is defined in this paper as estimating the 3D gaze direction parameterized by pitch and yaw, whereas the subsequent gaze-to-POR mapping represents the resulting POR in the 2D coordinate system of the calibrated scene plane. To map estimated 3D gaze direction to an attention position in the runway scene, a homography transformation is adopted as the core mapping model. As shown in Figure 4, given the estimated gaze angles, the corresponding normalized 2D gaze point is obtained by projecting the gaze direction onto a unit virtual plane: [tanϕ,tanθ]. This is equivalent to the projection of a unit gaze vector onto the image plane of a pinhole camera with unit focal length, consistent with standard gaze normalization conventions [13].

To establish the correspondence between the normalized gaze point and the runway scene coordinates, an individualized calibration procedure is performed prior to each session. N=16 calibration points with known scene coordinates ${\left\{p_{i}\right\}}_{i=1}^{N}$ are arranged on the runway reference plane, covering the full spatial extent of the viewing area. Participants sequentially fixate on each calibration point for 5 s; the stable central 3 s window is used to extract the corresponding normalized gaze coordinates $\left(u_{i},v_{i}\right)$, represented in homogeneous form as $q_{i}={\left[u_{i},v_{i},1\right]}^{T}$. This produces *N* matched pairs ${\left\{\left(q_{i},p_{i}\right)\right\}}_{i=1}^{N}$, where $p_{i}={\left[x_{i},y_{i},1\right]}^{T}$ denotes the homogeneous scene-level POR. The runway reference plane is modelled as a virtual canvas defined by four corner points on the physical floor or backdrop, establishing the pixel-coordinate system in which all POR outputs are expressed. The mapping between $q_{i}$ and $p_{i}$ is modelled as:

(18) $$\lambda_{i}p_{i}=Hq_{i},$$

where *H* is a 3×3 homography matrix and $\lambda_{i}$ is a non-zero homogeneous scale factor for the *i*-th correspondence. The homography matrix *H* was estimated from the calibration correspondences using the standard Direct Linear Transform formulation for planar projective transformations [33]. After calibration, *H* is applied online to convert each estimated gaze angle into the corresponding 2D scene gaze point.

### 2.5. Attention Analysis and Visualization

#### 2.5.1. Gaze Trajectory Smoothing via Adaptive Filtering

To suppress trajectory jitter and remove unreliable samples, the raw POR sequence is further processed through adaptive filtering, density clustering, and KDE. The parameter settings used in these processing stages were established through repeated preliminary pilot trials before the formal experiments and were subsequently held fixed across participants and experimental conditions.

The One Euro filter dynamically adjusts filtering intensity according to instantaneous gaze movement speed [34]. When the estimated POR velocity is low, the cutoff approaches $f_{c,\min}$, producing stronger smoothing of small fluctuations; as POR velocity increases, the cutoff increases so that the filter follows faster positional changes with reduced lag. Let the raw observation at time *t* be $x_{t}$, the filtered output be ${\hat{x}}_{t}$, the sampling interval be $\Delta t_{t}=t-t_{t-1}$, and the cutoff frequency be $f_{c}$. The smoothing coefficient α is determined by:

(19) $$\tau =\frac{1}{2\pi f_{c}},$$

(20) $$\alpha \left(f_{c},\Delta t_{t}\right)=\frac{1}{1+\frac{\tau }{\Delta t_{t}}}=\frac{2\pi f_{c}\Delta t_{t}}{2\pi f_{c}\Delta t_{t}+1}.$$

The recursive filtering equation is:

(21) $${\hat{x}}_{t}=\alpha \left(f_{c},\Delta t_{t}\right)\;\cdot \;x_{t}+\left[1-\alpha (f_{c},\Delta t_{t})\right]\;\cdot \;{\hat{x}}_{t-1}.$$

A high $f_{c}$ drives α→1 (rapid response); a low $f_{c}$ drives α→0 (strong smoothing). An independent low-pass filter applied to the first-order finite difference of the raw signal yields a smooth instantaneous rate estimate ${\hat{\dot{x}}}_{t}$:

(22) $${\hat{\dot{x}}}_{t}=\alpha \left(f_{c,d},\Delta t_{t}\right)\;\cdot \;\frac{x_{t}-{\hat{x}}_{t-1}}{\Delta t_{t}}+\left[1-\alpha (f_{c,d},\Delta t_{t})\right]\;\cdot \;{\hat{\dot{x}}}_{t-1},$$

where $f_{c,d}=1\;\mathrm{Hz}$ is the fixed derivative cutoff frequency. The main-signal cutoff frequency is then adapted linearly:

(23) $$f_{c}=f_{c,\min}+\beta \left|{\hat{\dot{x}}}_{t}\right|,$$

where ${\hat{\dot{x}}}_{t}$ is the filtered rate-of-change estimate; its magnitude is used so that the adaptive cutoff depends on the speed of signal variation rather than its direction. $f_{c,\min}=0.2\;\mathrm{Hz}$ is the minimum cutoff governing static smoothing strength and β=0.1 is the rate sensitivity coefficient. When $∥{\hat{\dot{x}}}_{t}∥$ is small, $f_{c}$ approaches $f_{c,\min}$, providing stronger smoothing of small-amplitude fluctuations; as $∥{\hat{\dot{x}}}_{t}∥$ increases, $f_{c}$ increases and the filter responds more rapidly to changes in the POR signal.

#### 2.5.2. DBSCAN Attention Point Clustering

Even after filtering, the POR sequence may contain outliers from blinks, sudden head movements, or model inference failures, which distort the gaze density distribution and create spurious attention hotspots. Therefore, DBSCAN was applied to the 2D POR coordinates to identify spatially dense clusters and classify isolated samples as noise [35]. The neighborhood radius and minimum neighborhood size were fixed at ε=40px and MinPts=30, respectively. All viewing-distance conditions were processed in the same scene-image coordinate system without distance-specific resizing or rescaling before DBSCAN or KDE. Thus, ε=40px represented the same neighborhood radius in image coordinates across conditions, whereas MinPts=30 was a sample-count criterion and therefore had no spatial or angular unit. Samples not assigned to a sufficiently dense cluster were treated as noise and excluded from subsequent analysis.

#### 2.5.3. Attention Heatmap Generation Based on KDE

To obtain a continuous probability density estimate of the spatial gaze distribution, KDE was applied to the post-clustering gaze samples retained by the processing pipeline ${\left\{p_{i}\right\}}_{i=1}^{n}$, where $p_{i}=\left(x_{i},\;y_{i}\right)$, to generate a smooth 2D probability density field. The attention density function at any scene location p=(x,y) is:

(24) $$\hat{f}\left(p\right)=\frac{1}{nh^{2}}\sum_{i=1}^{n}K\left(\frac{p-p_{i}}{h}\right),$$

where *n* is the number of gaze samples retained after filtering and clustering, *h* is the bandwidth parameter, and *K* is a 2D isotropic Gaussian kernel [36]. A fixed bandwidth of h=30px was used in the scene-image coordinate system. KDE was used only to visualize the spatial distribution of the processed gaze samples and did not enter AOI assignment or the cross-distance statistical analyses. The resulting density maps were normalized to [0, 1], mapped to a cool-to-warm pseudocolor scale, and overlaid on the corresponding runway images, with warmer colors indicating higher relative gaze density.

### 2.6. Prototype System Development

**Figure 5:**
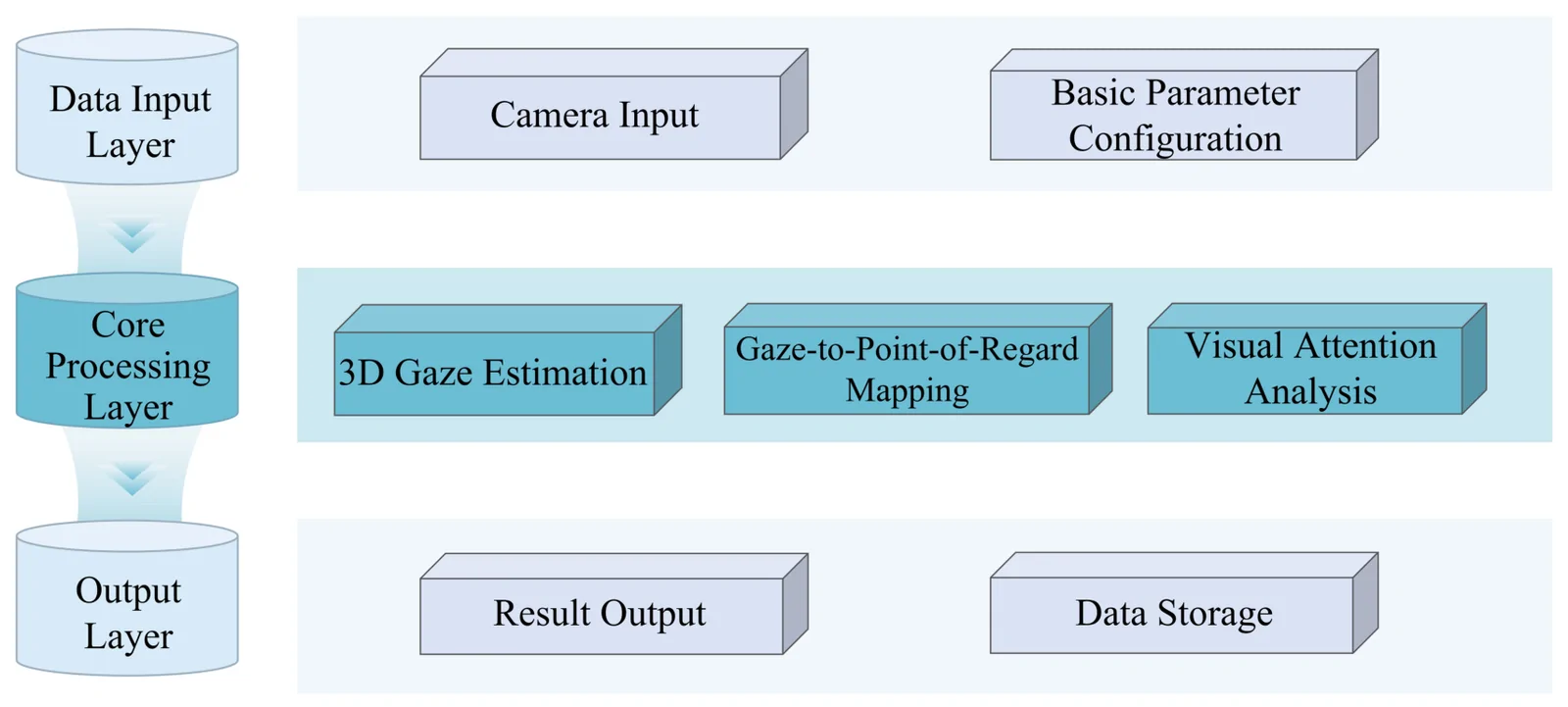
System overall architecture diagram.

As shown in Figure 5, the proposed framework is implemented as a prototype eye-tracking system for practical visual attention analysis in runway viewing scenarios. The system is developed in Python 3.9 and structured into three functional layers: a data input layer, a core computation layer, and a result output layer. The data input layer receives real-time camera video streams alongside auxiliary inputs including participant metadata, scene reference images, calibration grid configurations, and mapping parameters. The core computation layer implements the full algorithmic pipeline described in the preceding sections, comprising three parallel processing units responsible for 3D gaze estimation, gaze-to-POR mapping, and visual attention analysis. The result output layer supports both real-time visualization and post-session data export. During real-time operation, a live scene view is overlaid with the current POR position and gaze trajectory. After each session, the system exports POR sample sequences in CSV format, DBSCAN-based clustering results, and attention heatmaps.

**Figure 6:**
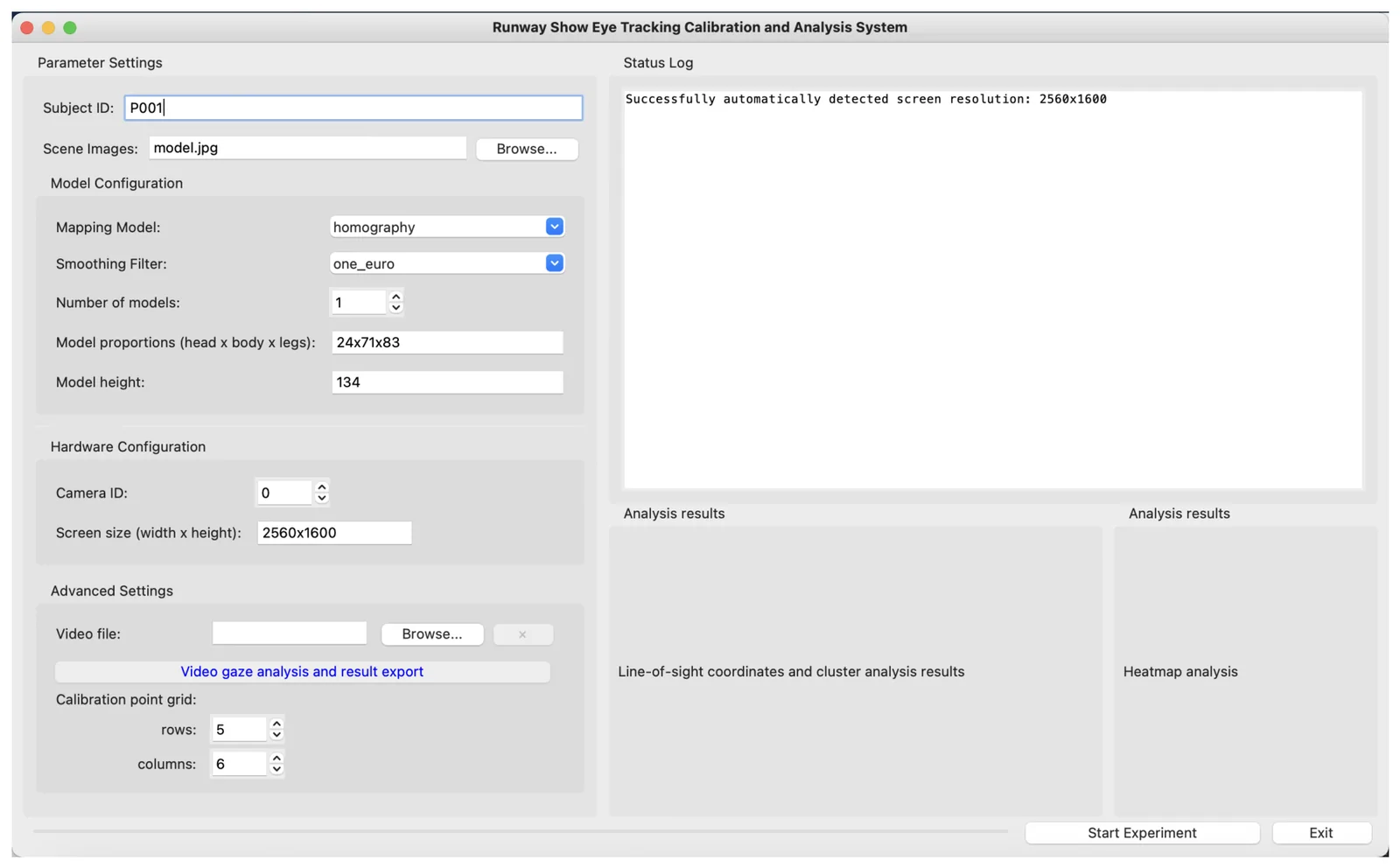
Interface diagram of the proposed system. Note. The displayed parameter values are illustrative; the experimental settings are specified in the Methods.

As shown in Figure 6, the system additionally provides a graphical configuration panel enabling key parameter adjustment without requiring code modification, supported by a scrollable status log for monitoring processing state. This interface allows researchers to operate the prototype without specialized programming expertise and supports application-oriented evaluation in simulated runway-observation settings.

### 2.7. Experimental Evaluation

To evaluate the proposed framework, two experiments were conducted. Experiment 1 benchmarked the proposed system against a commercial infrared eye tracker under controlled laboratory conditions to characterize post-calibration positional accuracy and spatial precision. Experiment 2 evaluated application-oriented AOI-localization feasibility in a simulated runway-observation scenario. Both experiments were executed on a Mac computer equipped with an Apple M2 chip (Apple Inc., Cupertino, CA, USA). For both experiments, the fixed pretrained gaze-estimation network described in Section 2.3.1 was used, while participant-specific calibration was performed only at the subsequent gaze-to-scene mapping stage, where the homography was estimated from calibration samples collected prior to each session.

#### 2.7.1. Experiment 1: Controlled Calibration-Grid Benchmark—Methods

(1) Participants and Benchmark setup

Experiment 1 involved 16 participants (8 males and 8 females; mean age ± SD, 24.8 ± 3.5 years). Participants were recruited through targeted online notices circulated within university and research-institute networks. Before enrollment, interested volunteers provided demographic and visual-status information together with information on their academic, research, or professional background relevant to the study context. Eligibility required prior involvement in at least one of the following activities: runway or fashion observation, aesthetic or design evaluation, fashion-content interpretation or dissemination, or related professional review and feedback. Eligibility was assessed from the information provided during this pre-enrollment screening according to the study-defined criteria; no separate occupational credential-verification procedure was used.

Eligibility also required a myopia refractive error not exceeding 3.00D in magnitude and the ability to resolve the experimental targets without corrective eyeglasses. To avoid reflections and partial occlusion in the RGB eye images, no participant wore eyeglasses during the experiment. Written informed consent was obtained from all participants.

**Figure 7:**
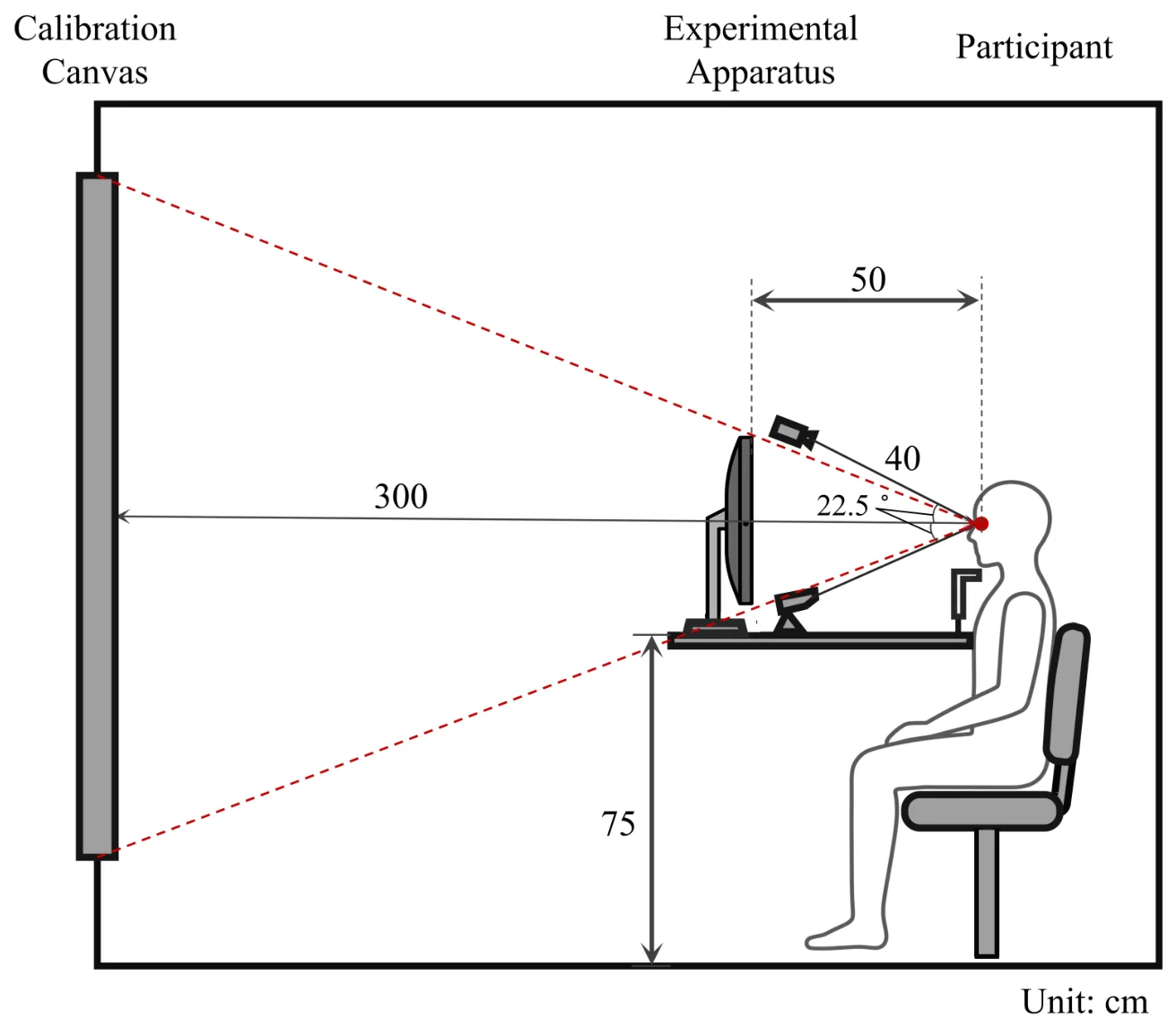
Spatial layout of Experiment 1.

The benchmark device was the EyeLink 1000 Plus, a desktop-mounted video-based pupil-corneal-reflection eye tracker used as the laboratory reference comparator for the RGB-webcam system [37,38]. Video of the participant’s face and eye regions was acquired using a USB RGB webcam labeled “HD WEBCAM”. During experimental data acquisition, the camera operated at 1920×1080 px and an achieved frame rate of 30 fps. As shown in Figure 7, the participant sat at a table (height: 75 cm), with head position stabilized using a chin rest (height: 25 cm). The chin rest was used only in Experiment 1 to minimize head-motion-related variability and to provide a controlled common geometry for the within-protocol comparison with the EyeLink 1000 Plus; it is not a hardware requirement of the proposed system.

**Figure 8:**
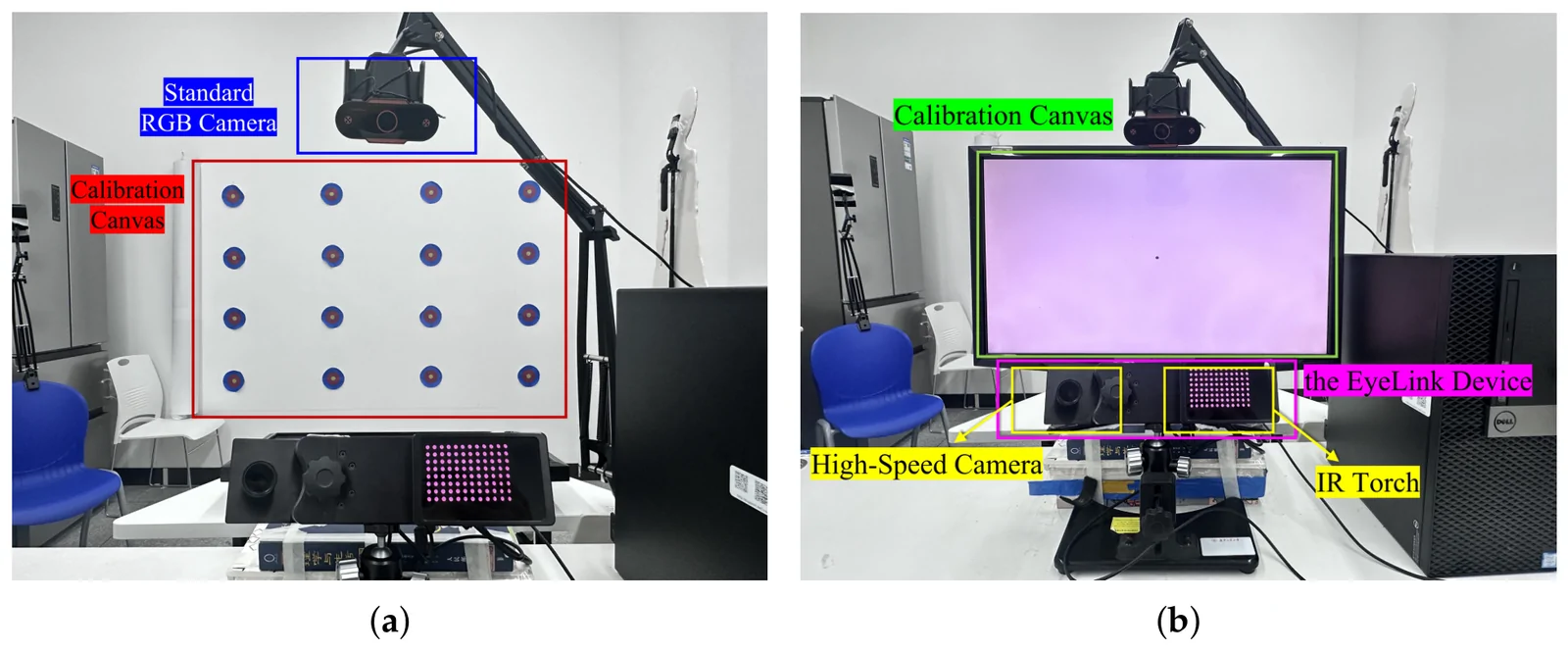
Experimental setup of the two systems: (**a**) Proposed system; (**b**) EyeLink 1000 Plus.

A 23-inch monitor (50.9 × 28.6 cm) was positioned 50 cm from the eyes. The EyeLink infrared unit, equipped with a 35-mm lens for head-stabilized tracking, was positioned 22.5° below the participant’s line of sight at a 40 cm camera-to-eye distance. The experimental task required fixation only at the 16 inset target locations, with maximum target eccentricities of approximately 19.0° horizontally and 12.1° vertically. This configuration was used as the study-specific benchmark geometry for the EyeLink system. The proposed system’s cameras were symmetrically mounted 22.5° above at the same distance. This symmetric placement was used to reduce differences in nominal camera-angle offset between the two acquisition configurations. Figure 8 shows the experimental setup of the two systems.

(2) Calibration and Benchmark Tasks

**Figure 9:**
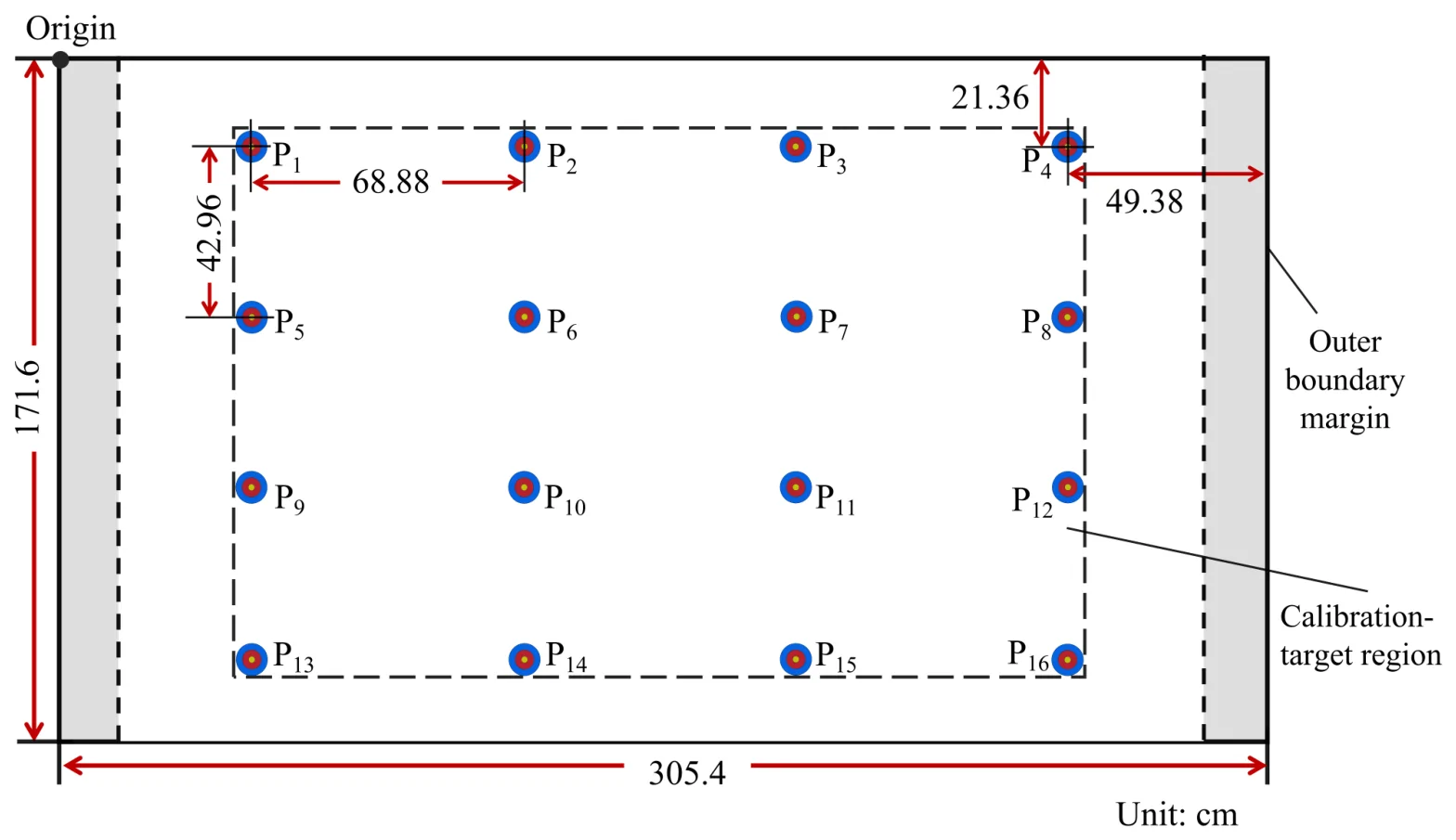
Wall canvas and 16-point distribution map for Experiment 1.

**Figure 10:**
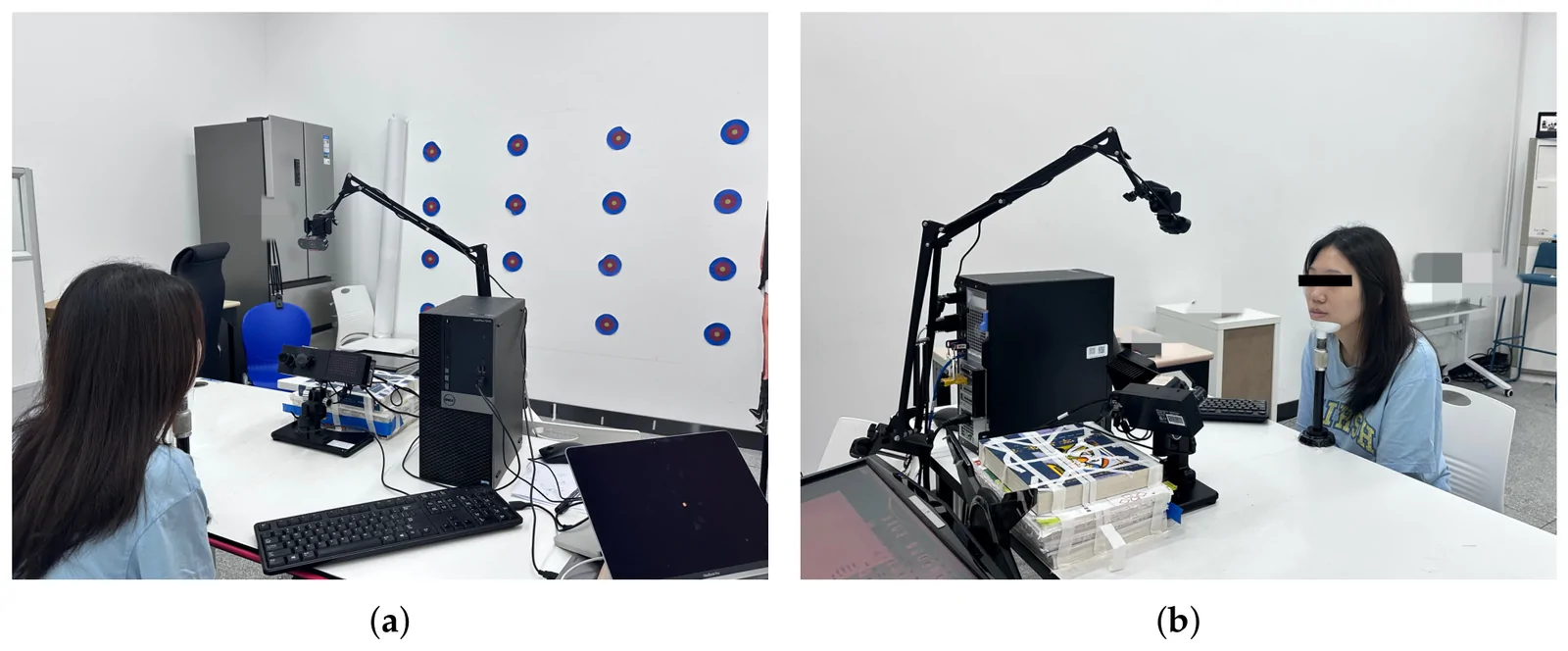
Procedure record of Experiment 1: (**a**) Back view; (**b**) Front view.

Because the EyeLink 1000 Plus performs gaze prediction on the monitor plane while the proposed system maps gaze to a calibrated scene plane, a unified comparison framework was established. A rectangular calibration canvas (305.4 × 171.6 cm) was mounted on a wall 3 m from the participant’s eyes, with its center aligned with the monitor center along the participant’s horizontal line of sight (Figure 7). The physical canvas was represented by a 1920×1080 virtual coordinate system, corresponding to approximately 0.159cm/px in both horizontal and vertical directions. Canvas dimensions were derived from monitor dimensions by a proportional scaling factor of 6, ensuring that corresponding points on the monitor and canvas subtended identical visual angles and thus corresponded to the same 3D gaze direction. Sixteen evenly distributed target points were marked on both surfaces, as shown in Figure 9. The same 16 spatial target locations were used for participant-specific calibration and subsequent evaluation. Although the spatial target locations themselves were not held out, the evaluation samples were acquired in a separate post-calibration fixation recording after *H* had been estimated and were not used to fit the homography. Accordingly, $A_{\mathrm{pos}}$ quantifies target-referenced post-calibration localization error at previously calibrated locations. During evaluation, participants again fixated each target for 5 s; the central 3 s window was used for metric computation. Figure 10 shows the experimental procedure. For each participant, the proposed system and EyeLink were evaluated in separate recordings, with the order of the two system measurements randomized across participants. The two systems used the same metric definitions while retaining their native processing pipelines. EyeLink recorded right-eye gaze at 2000 Hz with filter setting 2, and was analyzed from its native output. For the proposed system, $A_{\mathrm{pos}}$ and $A_{\mathrm{spa}}$ were calculated from the POR output after the operational One Euro filtering and DBSCAN stages. The tracker-recorded validation summaries yielded a mean validation error of 0.67±0.19° (range, 0.29–0.97°) and a mean maximum validation error of 3.59±1.51° (range, 1.55–7.37°). For both systems, $A_{\mathrm{pos}}$ and $A_{\mathrm{spa}}$ were calculated separately at each target and equally averaged across the 16 targets to obtain participant-level values.

(3) Evaluation Indicators

Target-referenced localization error was quantified by $A_{\mathrm{pos}}$ (px), defined as the mean Euclidean distance between each estimated gaze sample and the corresponding target position:

(25) $$A_{\mathrm{pos}}=\frac{1}{N}\sum_{i=1}^{N}{∥g_{i}-t_{i}∥}_{2}.$$

$A_{\mathrm{pos}}$ is a combined localization-error measure that reflects both systematic offset and sample dispersion. It is therefore not directly comparable to validation or manufacturer-specified accuracy figures defined as the offset of the mean gaze position from the target.

Spatial precision was quantified by the root-mean-square (RMS) spatial-dispersion metric $A_{\mathrm{spa}}$ (px), defined as the two-dimensional RMS distance of the gaze samples from their centroid:

(26) $$A_{\mathrm{spa}}=\sqrt{\frac{1}{N}\sum_{i=1}^{N}{∥g_{i}-\bar{g}∥}_{2}^{2}},$$

Here, $g_{i}$ and $t_{i}$ denote the 2D gaze and target positions, respectively, and $\bar{g}$ denotes the centroid of the gaze samples within the same target interval. For the proposed system, $A_{\mathrm{spa}}$ was calculated from POR samples available after the fixed operational One Euro filtering and DBSCAN stages. Accordingly, $A_{\mathrm{spa}}$ characterizes the spatial precision of the processed end-to-end POR output. Lower $A_{\mathrm{pos}}$ indicates lower combined localization error, whereas lower $A_{\mathrm{spa}}$ indicates higher spatial precision.

Because both metrics are expressed in pixels and therefore depend on the coordinate scale, participant-level $A_{\mathrm{pos}}$ and $A_{\mathrm{spa}}$ values were additionally converted to physical distance and degrees of visual angle. All cross-system metrics were computed in a common 1920×1080 coordinate system. Proposed-system coordinates, originally represented in a 2560×1600 scene frame, were transformed according to $x_{c}=x\left(\frac{1920}{2560}\right)\;\mathrm{and}\;y_{c}=y\left(\frac{1080}{1600}\right)$. EyeLink coordinates were retained in their native 1920×1080 frame. The 1920×1080 virtual canvas represented a physical area of 305.4 × 171.6 cm, yielding an approximately isotropic scale of 0.159cm/px. Pixel distances were therefore converted as

(27) $$d_{\mathrm{cm}}=d_{\mathrm{px}}\frac{305.4}{1920}.$$

Given the D=300 cm eye-to-canvas distance, the corresponding visual angle was calculated as

(28) $$\theta_{\deg}=2\arctan\left(\frac{d_{\mathrm{cm}}}{2D}\right)\frac{180}{\pi },\;D=300\;\mathrm{cm}.$$

The angular values are reported to facilitate interpretation relative to conventional eye-tracking literature. The Experiment 1 proposed-system metrics reported in this study were calculated from the POR sequences after the operational filtering and DBSCAN stages and therefore characterize the processed end-to-end system output rather than the unfiltered gaze-estimation signal.

#### 2.7.2. Experiment 2: Simulated Runway Observation

(1) Participants and Runway-Observation Setup

**Figure 11:**
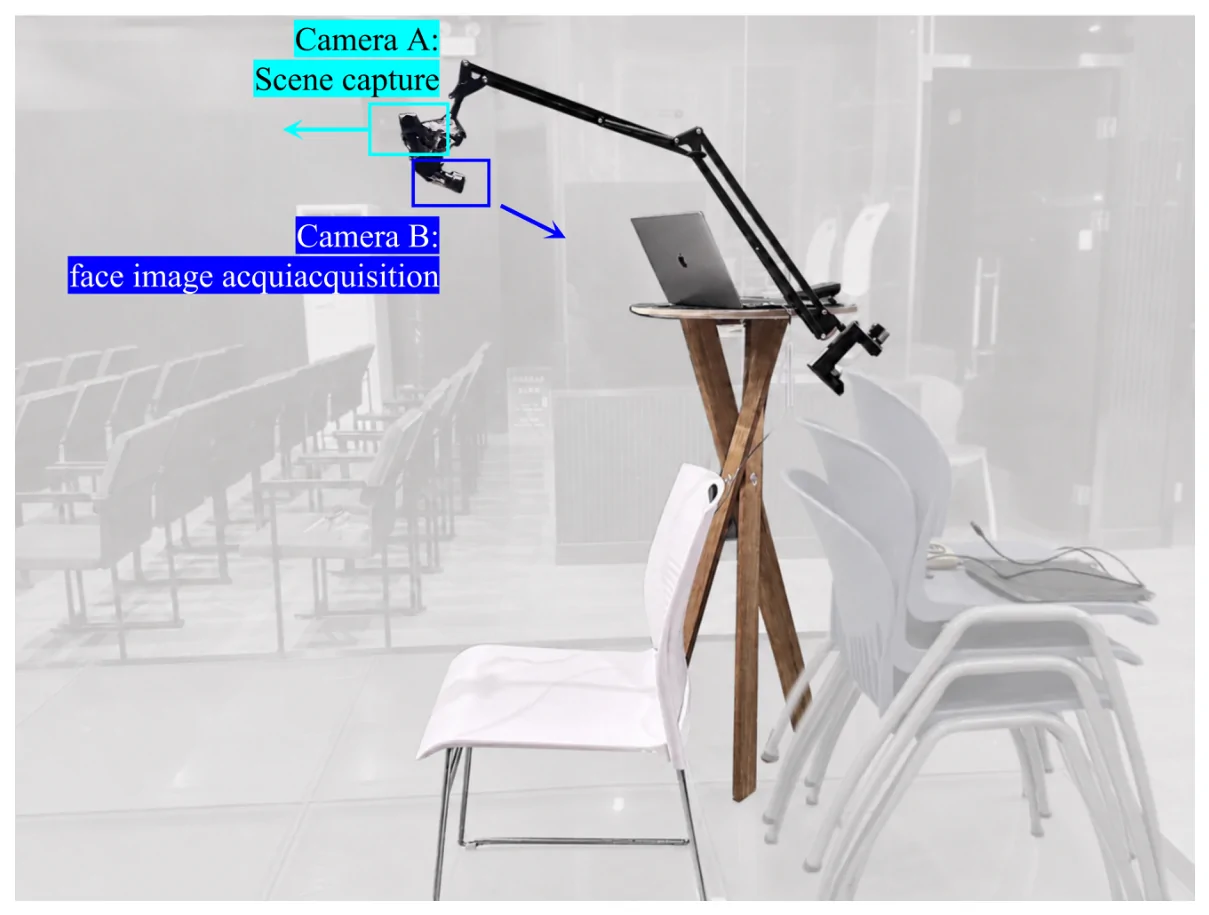
Device layout diagram for Experiment 2.

Experiment 2 applied the proposed system to a simulated runway observation scenario. A separate group of 16 participants (8 males and 8 females; mean age ± SD, 26.4 ± 3.0 years) who had not participated in Experiment 1 was recruited using the same targeted online recruitment, pre-enrollment screening procedure, and eligibility and exclusion criteria as in Experiment 1. Video of the participants’ face and eye regions was acquired using the same USB RGB webcam labeled “HD WEBCAM” as in Experiment 1, at a resolution of 1920×1080 px and an achieved frame rate of 30 fps. Unlike Experiment 1, no chin rest or other mechanical head-stabilization device was used in Experiment 2. Participants were allowed to maintain their natural head posture and make small spontaneous head movements during observation, while the frame-wise face and eye localization and eye-region normalization stages continuously updated the eye regions used for gaze estimation. The experimental layout is shown in Figure 11.

(2) Calibration and Observation Tasks

For Experiment 2, the observation distances were determined according to the spatial characteristics of runway viewing. Considering that the minimum social distance between runway models and KOLs is typically 2 m during a performance, the nearest observation point was set at a distance of 2 m from the participants’ eyes. Based on this reference distance, multiples of 2 m were defined as two additional observation nodes in the direction of the line of sight along the runway: 4 m and 6 m, corresponding to the medium-distance and long-distance observation phases, respectively. A calibration canvas was positioned at 7 m, 1 m beyond the farthest observation node, so that the physical calibration surface did not obstruct the subsequent 6 m, 4 m and 2 m approach path. During calibration, each participant fixated the 16 evenly distributed calibration points sequentially for 5 s, with the central 3 s window used for homography estimation. The resulting participant-specific homography was retained across all three observation distances to maintain a single-calibration condition during the continuous approach. Because the target subsequently occupied depths of 6 m, 4 m and 2 m, its increasing departure from the calibration plane could introduce depth-dependent POR misregistration, particularly at the 2 m condition. This changing-depth configuration was retained to approximate the approach trajectory of a runway model.

**Figure 12:**
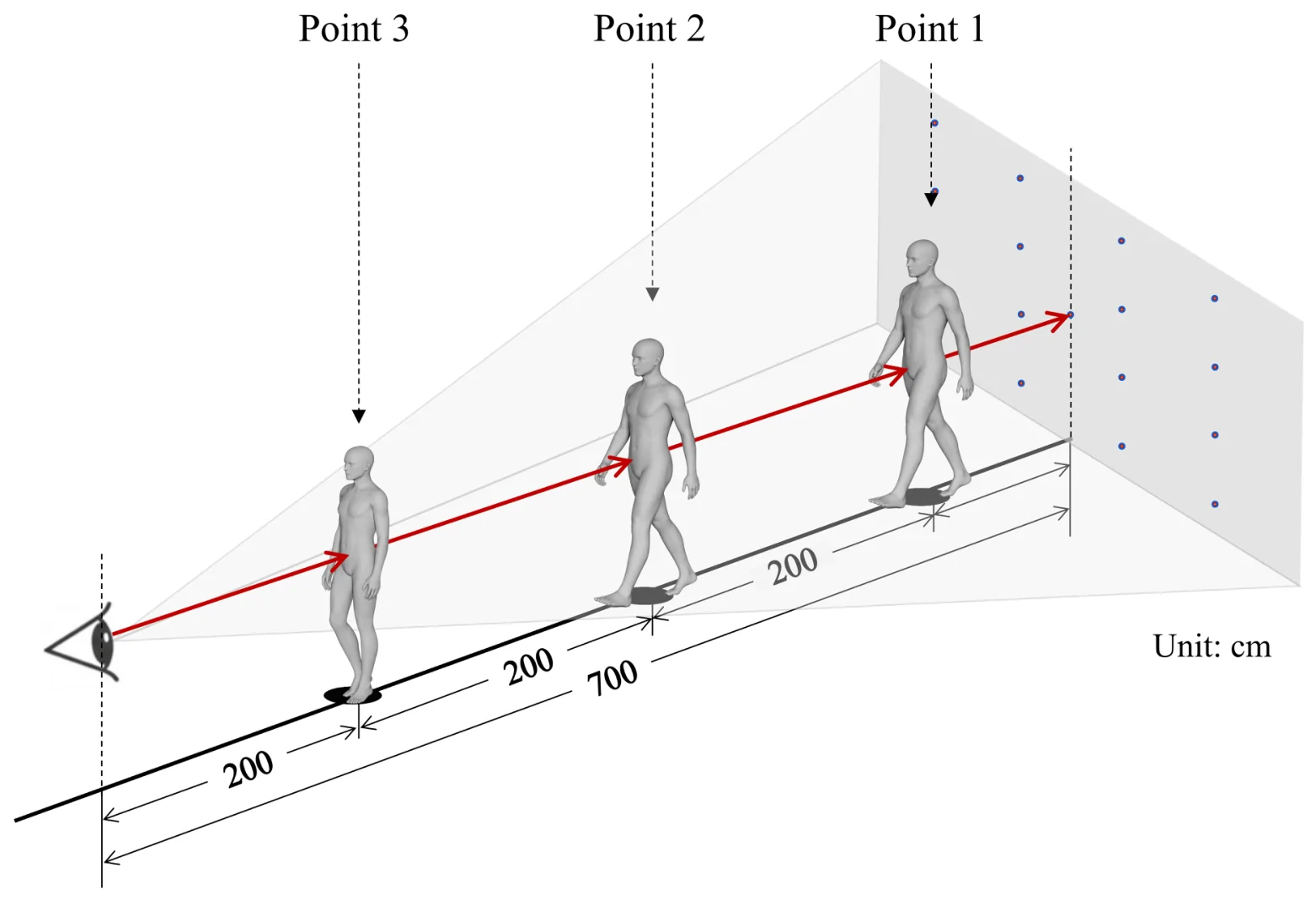
Schematic diagram of the observation task in Experiment 2. Note. The red arrows indicate the schematic viewing direction along the observation axis. The colored dots on the reference canvas mark the calibration points. Points 1, 2, and 3 correspond to viewing distances of 6, 4, and 2 m, respectively.

**Figure 13:**
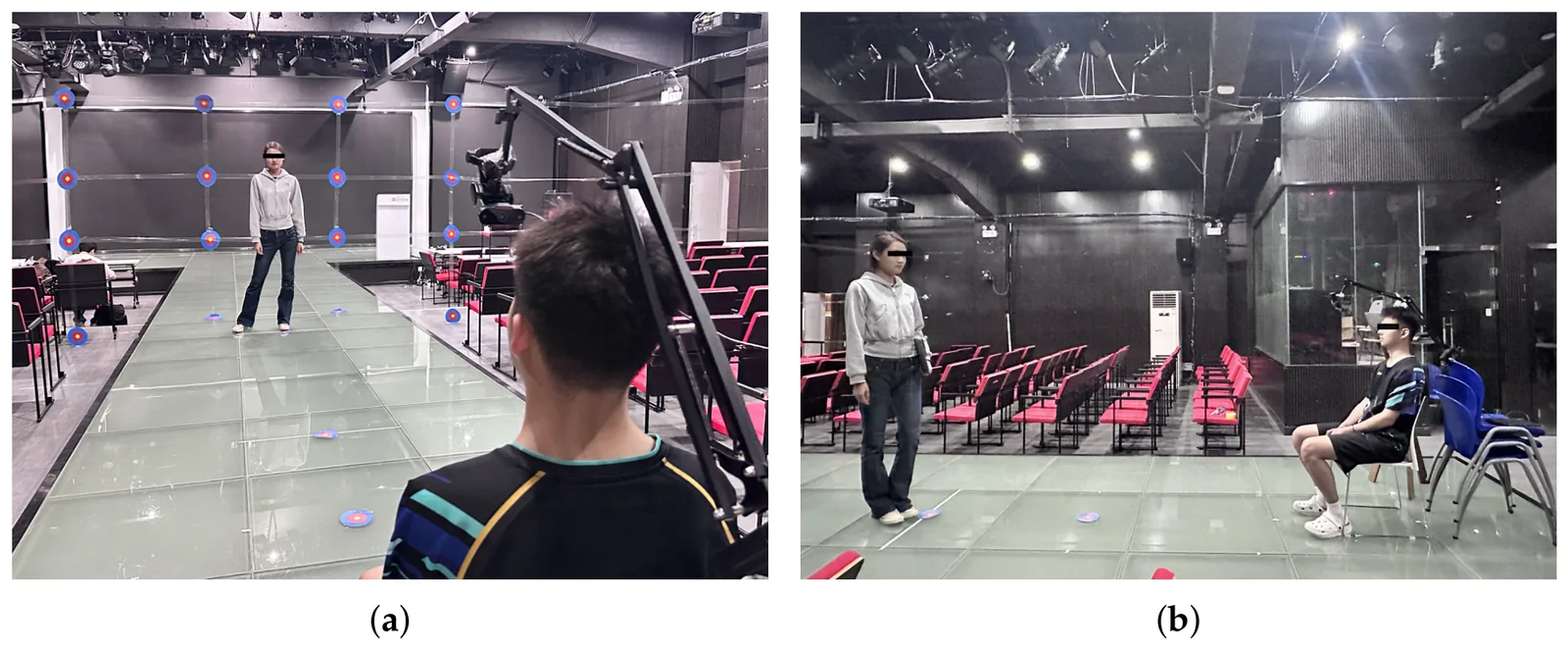
Experiment record of Experiment 2: (**a**) Front view; (**b**) Side view.

In the formal observation task, to simulate the walking trajectory of a runway model towards the audience, a trained experimental assistant played the role of a model, continuously walking towards the participants from in front of the canvas. Along the walking path, locations 6 m, 4 m and 2 m from the participants’ eyes were designated as Points 1, 2, and 3, respectively. The assistant walked through these nodes without pausing. Each 2 m interval took approximately 5–8 s, corresponding to an overall approach duration of approximately 10–16 s from 6 m to 2 m. Because the distance between the experimental assistant and the participants changed continuously during the walking process, the size of the model’s projection in the scene image also changed accordingly. As shown in Figure 12, keyframes were extracted from the video recording when the experimental assistant was at points 1, 2, and 3, allowing for a comparison of the gaze results under different distance conditions with a unified spatial reference. POR samples corresponding to the three predefined observation conditions were analyzed separately for AOI localization and visualization. Participants were instructed to attend to the model’s right-chest region. No unique point-level ground-truth coordinate was specified or recorded. The right-chest region therefore served as the predefined target AOI for evaluating whether the estimated gaze remained spatially concentrated as viewing distance changed. This region was selected because it contains salient garment details such as breast pockets and lapel stitching. Figure 13 shows a representative experimental record.

(3) AOI Definition and Evaluation Measures

**Figure 14:**
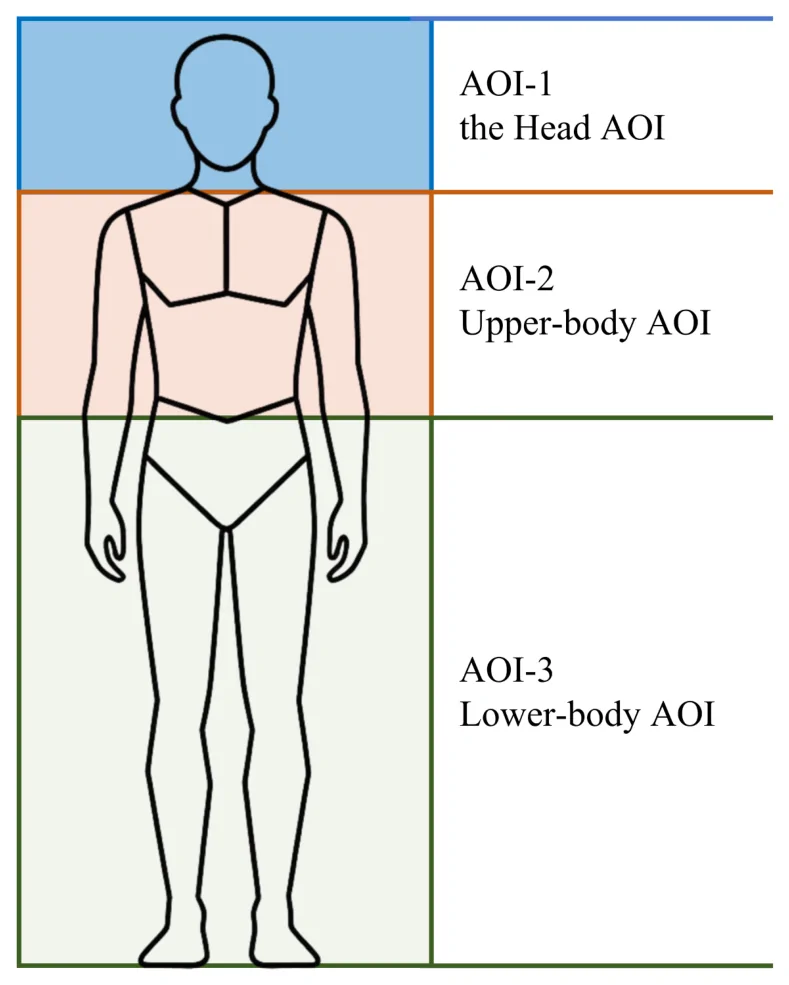
Body-region AOI definitions for the frontal-view runway target.

For AOI analysis, the visible body of the model in each distance-specific scene frame was partitioned into three mutually exclusive semantic body regions (Figure 14): AOI-1 (head: face and hairstyle), AOI-2 (upper body: torso and garment details), and AOI-3 (lower body: trousers and footwear). The AOIs followed the visible anatomical and garment boundaries in each scene; their image extents therefore varied with the projected size and position of the model. Samples falling outside all three AOIs were classified as off-target.

To quantify the distance-dependent change in AOI extent, the body-region templates comprised a 25×36 cm head-and-neck region, a 44×64 cm upper-body region, and a 44×77 cm lower-body region. The upper- and lower-body widths corresponded to the measured shoulder width of 42 cm plus a 1 cm margin on each side, and the three regional heights summed to the model’s height of 177 cm. For parallel body and reference planes, the projected dimensions on the 7 m reference plane scaled with viewing distance. The corresponding projected AOI extents in the 2560×1600 reference coordinate system and their angular extents at each viewing distance are reported in Table 1. As expected from perspective projection, all three AOIs increased in both image extent and visual angle as viewing distance decreased, with the largest extents occurring at 2 m.

For each participant and viewing distance, $N_{\mathrm{proc}}$ denotes the valid POR samples available after the gaze-processing pipeline and before AOI assignment. Samples assigned to the three non-overlapping AOIs constitute $N_{\mathrm{retained}}=\sum_{j=1}^{3}N_{\mathrm{AOI},j}$, whereas samples outside all AOIs constitute $N_{\mathrm{off}}$; therefore, $N_{\mathrm{proc}}=N_{\mathrm{retained}}+N_{\mathrm{off}}$. The AOI-retained sample percentage was calculated as

(29) $$R_{\mathrm{retained}}=\frac{N_{\mathrm{retained}}}{N_{\mathrm{proc}}}\times 100\%.$$

For AOI *j*,

(30) $$P_{\mathrm{AOI},j}=\frac{N_{\mathrm{AOI},j}}{N_{\mathrm{proc}}}\times 100\%.$$

### 2.8. Statistical Analysis

Participant-level paired data were analyzed using nonparametric methods to avoid relying on normality assumptions with the sample size available in each experiment. All tests were two-sided, and the significance level was set at α=0.05. Descriptive statistics are reported as mean ± standard deviation together with participant-level values.

For Experiment 1, between-system comparisons were performed using two-sided exact Wilcoxon signed-rank tests on participant-level paired measurements. Rank-biserial correlation was reported as the paired effect-size measure. Mean paired differences were calculated as proposed-system minus EyeLink values, and 95% confidence intervals were estimated using participant-level percentile bootstrap resampling with 200,000 resamples while preserving the paired structure. For target-level analysis, Bland–Altman agreement was assessed across the 16 paired target-position means, and target dependence of the participant-level between-system differences was tested using Friedman tests, with Kendall’s *W* reported as the effect-size measure.

For Experiment 2, Friedman tests were used to examine the effect of viewing distance on (1) AOI-retained sample percentage and (2) the percentage of post-processing gaze samples assigned to the upper-body AOI. When a Friedman omnibus test was statistically significant, exact pairwise Wilcoxon signed-rank tests were conducted, and the resulting *p*-values were adjusted using the Holm procedure. Kendall’s *W* was reported as the effect-size measure for Friedman tests, and rank-biserial correlation was reported for pairwise Wilcoxon comparisons. Statistical analyses were conducted in Python 3.9.

## 3. Results

### 3.1. Experiment 1: Controlled Calibration-Grid Benchmark—Results

Per-participant benchmark results for the EyeLink 1000 Plus and the proposed system are presented in Table 2. After coordinate harmonization, all pixel-based measurements were expressed in a common 1920×1080 coordinate system, and the corresponding visual angles were calculated using the same experimental geometry. For positional error, the proposed system yielded a mean $A_{\mathrm{pos}}$ of 82.19±8.37 px (2.50±0.25°), compared with 57.18±18.10 px (1.74±0.55°) for EyeLink. The mean paired difference was 25.01 px (0.76°), with a 95% bootstrap confidence interval of 17.25–32.66 px (0.52°–0.99°). The difference was significant (W=2, p<0.001, $r_{\mathrm{rb}}=0.971$). For the processed-output spatial-precision measure $A_{\mathrm{spa}}$, the proposed system yielded a larger value than EyeLink under the present benchmark. Mean $A_{\mathrm{spa}}$ was 76.21±7.90 px (2.31±0.24°) for the proposed system and 33.64±12.21 px (1.02±0.37°) for EyeLink. The mean paired difference was 42.57 px (1.29°), with a 95% bootstrap confidence interval of 35.86–49.64 px (1.09°–1.51°), and the difference was significant (W=0, p<0.001, $r_{\mathrm{rb}}=1.000$).

Target-level analysis further showed that the positional-error difference was not spatially uniform. Across the 16 target positions, the mean proposed-minus-EyeLink bias in $A_{\mathrm{pos}}$ was +25.01 px (+0.76°), with Bland–Altman limits of agreement from −40.29 to +90.31 px (−1.22° to +2.74°). The between-system difference varied significantly across target locations ($\chi^{2}\left(15\right)=84.40$, p<0.001, Kendall’s W=0.352), indicating a target-dependent component in positional accuracy. In contrast, the between-system difference in $A_{\mathrm{spa}}$ was more consistent across the target grid. The mean $A_{\mathrm{spa}}$ bias was +42.57 px (+1.29°), with limits of agreement from +23.19 to +61.94 px (+0.70° to +1.88°), and no significant target-position effect was detected ($\chi^{2}\left(15\right)=20.06$, p=0.170, Kendall’s W=0.084).

Although the proposed system showed lower positional accuracy and spatial precision than the laboratory-grade EyeLink reference, its mean $A_{\mathrm{pos}}$ and $A_{\mathrm{spa}}$ remained within approximately 2.5° and 2.3°, respectively. Moreover, the relatively small between-participant variability of the proposed system (SD=0.25° for $A_{\mathrm{pos}}$ and SD=0.24° for $A_{\mathrm{spa}}$) indicates stable performance across participants. These results suggest that the system retains useful spatial localization capability under controlled conditions, particularly for applications in which gaze is interpreted at the level of broader regions of interest.

### 3.2. Experiment 2: Performance Across Viewing Distances

Per-participant results for the 6 m, 4 m and 2 m viewing conditions are reported in Table 3, Table 4 and Table 5. The mean AOI-retained sample percentages were 95.96±4.10% at 6 m, 97.31±3.50% at 4 m, and 80.43±14.95% at 2 m. Participant-level AOI-retained sample percentages ranged from 84.9% to 100.0% at 6 m, from 88.1% to 100.0% at 4 m, and from 54.6% to 100.0% at 2 m. The sample counts further show that the reduction at 2 m was associated with a markedly larger number of post-processing POR samples classified as off-target. Across the 16 participants, $N_{\mathrm{off}}$ totaled 746 of 3681 processed samples at 2 m, compared with 99 of 3618 at 4 m and 142 of 3524 at 6 m.

The small nonzero lower-body percentages may reflect brief gaze shifts away from the instructed right-chest target; however, the present data cannot determine whether these samples represent actual gaze shifts or occasional localization error.

A Friedman test indicated a significant effect of viewing distance on the AOI-retained sample percentage ($\chi_{F}^{2}\left(2\right)=9.50$, p=0.009, Kendall’s W=0.30). Holm-adjusted pairwise Wilcoxon signed-rank tests showed higher AOI-retained sample percentages at 6 m than at 2 m (W=8, $p_{\mathrm{adj}}=0.002$, $r_{\mathrm{rb}}=0.88$) and at 4 m than at 2 m (W=10, $p_{\mathrm{adj}}=0.003$, $r_{\mathrm{rb}}=0.85$). No statistically significant difference was detected between the 6 m and 4 m conditions (W=55, $p_{\mathrm{adj}}=0.528$, $r_{\mathrm{rb}}=-0.19$).

The mean percentages of post-processing gaze samples $N_{\mathrm{proc}}$ assigned to the upper-body AOI were 83.9%, 66.9%, and 75.9% at 6 m, 4 m, and 2 m, respectively. A Friedman test did not detect a statistically significant difference in upper-body AOI assignment percentage across the three viewing distances ($\chi_{F}^{2}\left(2\right)=4.50$, p=0.105, Kendall’s W=0.14). Thus, although the proportion of post-processing gaze samples falling within the predefined AOIs varied significantly with viewing distance, the upper-body AOI assignment percentage did not show a statistically detectable cross-distance difference.

**Figure 15:**
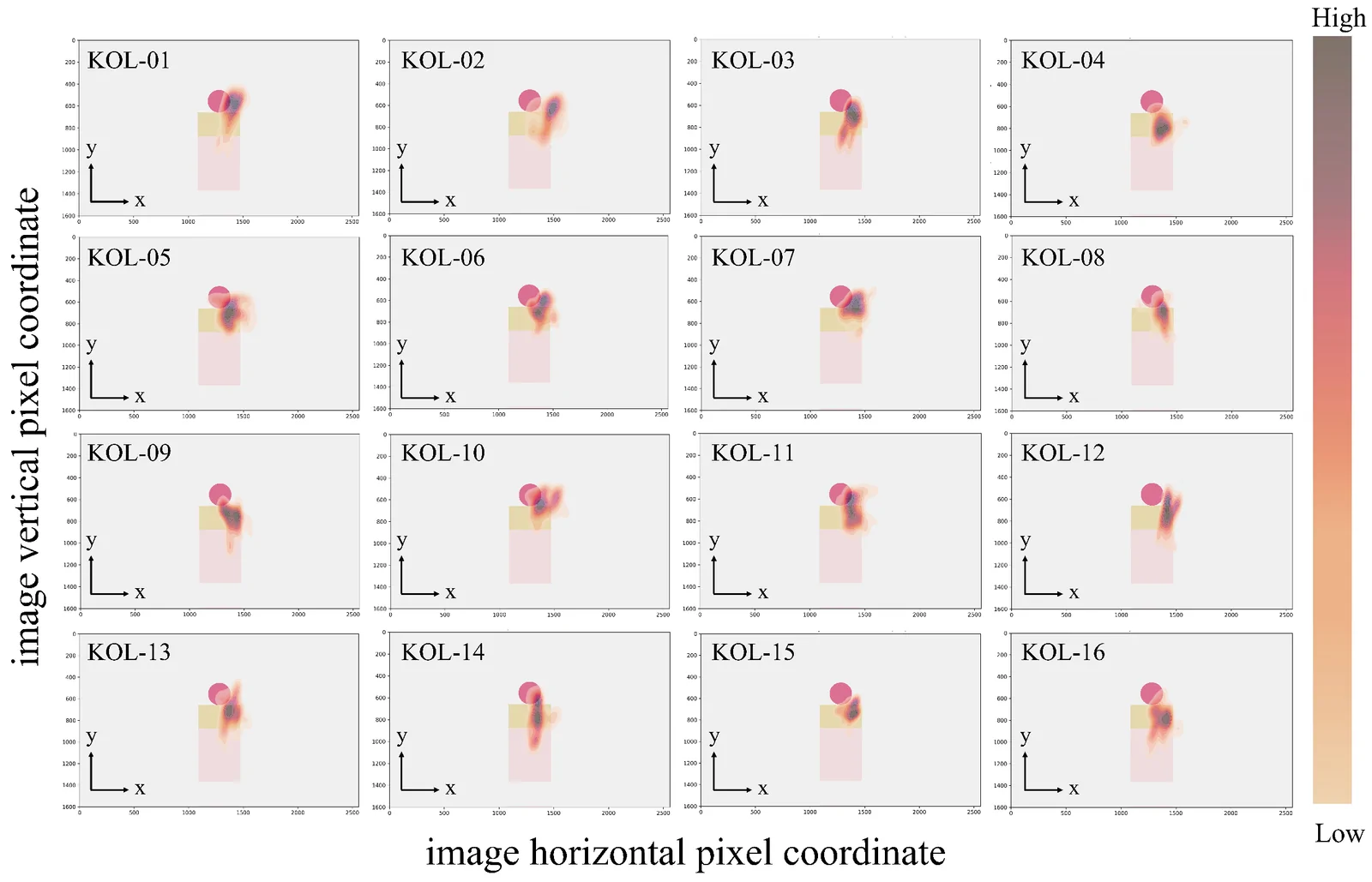
Gaze heatmap under the 6 m condition. Note. The body-region representation reflects the scene view at this distance; only the portions within the displayed image area are shown.

**Figure 16:**
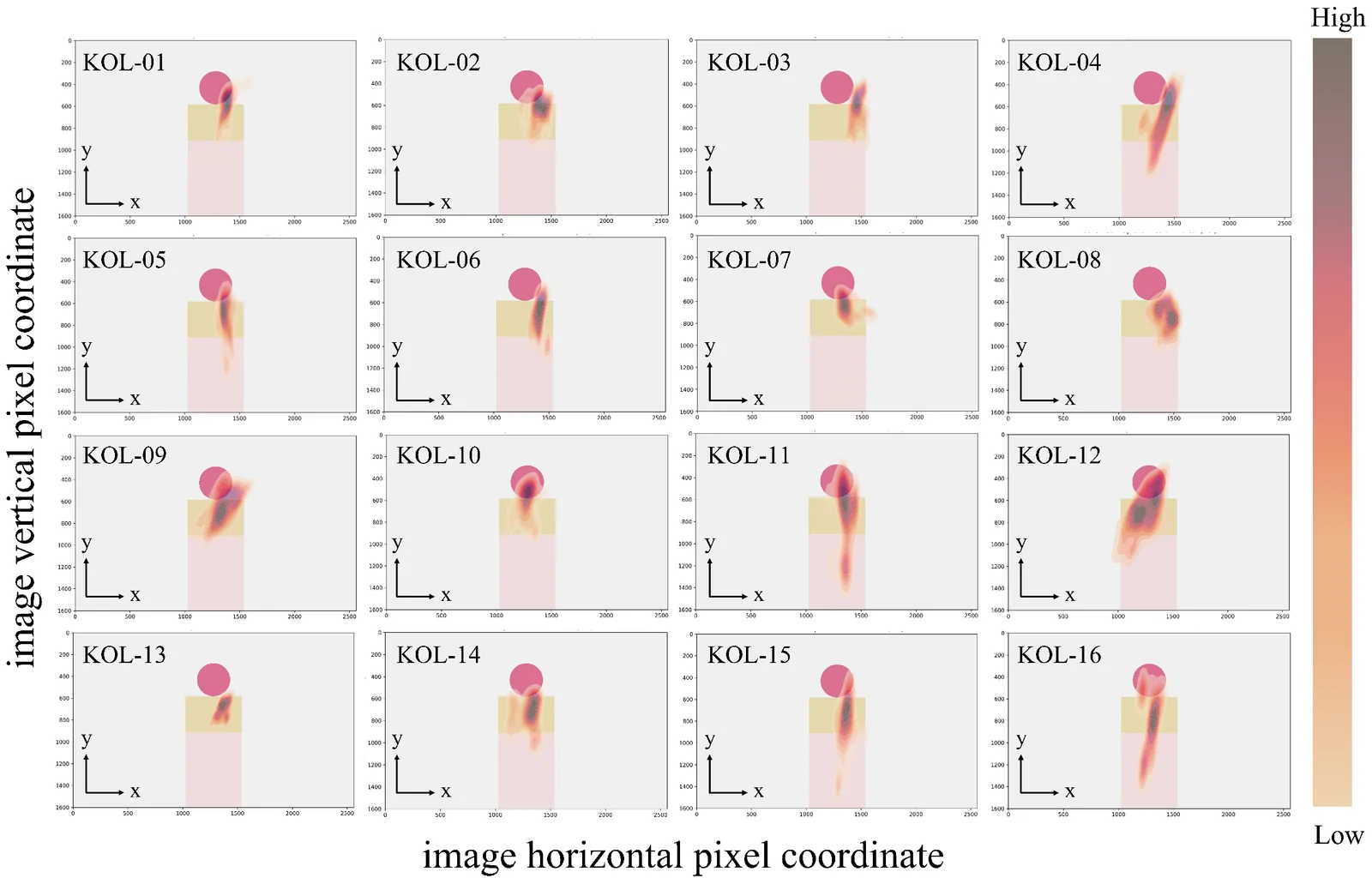
Gaze heatmap under the 4 m condition. Note. The body-region representation reflects the scene view at this distance; only the portions within the displayed image area are shown.

**Figure 17:**
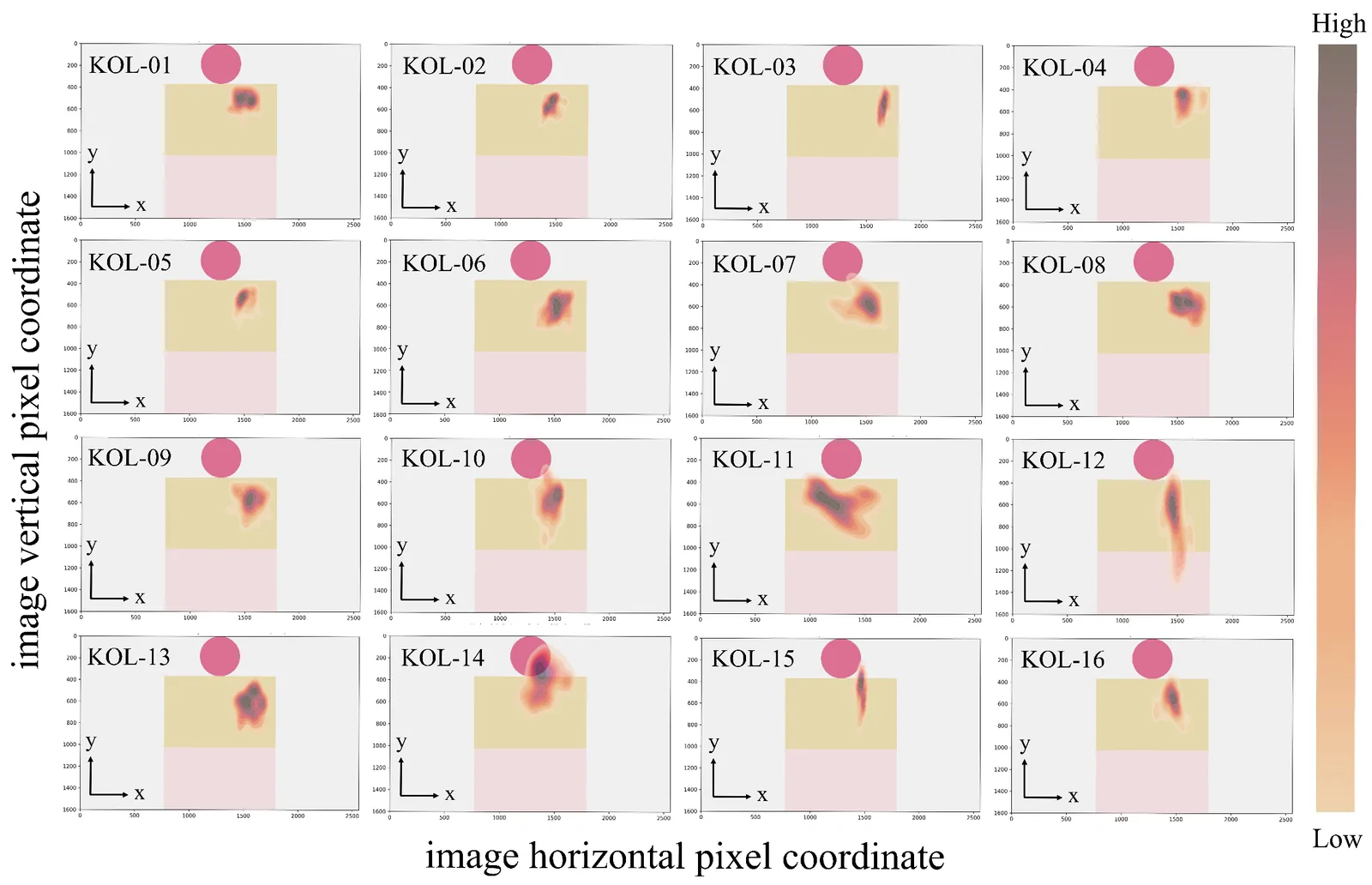
Gaze heatmap under the 2 m condition. Note. The body-region representation reflects the scene view at this distance; only the portions within the displayed image area are shown.

The KDE gaze-density maps are shown in Figure 15, Figure 16 and Figure 17. Across participants, the highest-density regions were predominantly located within the upper-body AOI and were concentrated around the predefined right-chest observation target. This spatial concentration was observed at 6 m, 4 m, and 2 m, although the distribution and AOI-retained sample percentages varied among participants and across viewing distances.

## 4. Discussion

### 4.1. General Findings

Experiment 1 provided a controlled characterization of the post-calibration target-localization performance of the complete RGB-to-scene gaze-mapping pipeline under the fixed 3 m benchmark geometry. Although the proposed system showed higher positional error than the EyeLink 1000 Plus, its mean error remained within a low-single-digit visual-angle range (2.50° vs. 1.74°). This benchmark therefore provided a study-specific performance reference for the subsequent application-oriented AOI-level evaluation. Because Experiment 2 used different 2, 4, and 6 m viewing geometries and did not obtain independent point-level ground truth, the Experiment 1 results should not be interpreted as positional-accuracy estimates for those conditions.

The observed performance gap is consistent with the different sensing and estimation architectures of the two systems. The proposed framework derives scene-level POR from passive RGB imagery through eye-region normalization, gaze-direction estimation, individualized scene mapping, and downstream POR processing, allowing uncertainty from multiple stages to propagate to the final gaze estimate. This limits fine point-of-gaze discrimination; accordingly, Experiment 2 focused on localization within predefined anatomical AOIs rather than fine point locations.

Experiment 2 further examined the system through an application-oriented AOI-localization feasibility test under viewing conditions designed to approximate runway observation. AOI-retained sample percentages remained high at 6 m and 4 m but decreased at 2 m, indicating reduced AOI-level localization performance at the closest viewing distance. Importantly, this reduction occurred despite the AOIs having their largest projected and angular extents at 2 m. For example, the upper-body AOI increased from approximately 246×358 px (4.20°×6.11°) at 6 m to 737×1074 px (12.55°×18.18°) at 2 m. Thus, the lower AOI-retained sample percentage at 2 m cannot be explained by a smaller projected target or a smaller AOI acceptance region.

At 2 m, the reduction in AOI-retained sample percentage was accompanied by a marked increase in $N_{\mathrm{off}}$, indicating that more post-processing POR samples fell outside the predefined AOIs. A plausible contributor is the changing-depth geometry relative to the single-plane calibration. As the target moved progressively farther from the calibration plane, depth-dependent mismatch between the mapped POR coordinates and the distance-specific AOI boundaries may have increased, resulting in more samples being classified as off-target at the closest viewing distance. A further plausible contributor is target motion relative to the static keyframe AOIs. For an approximately constant-speed approach along the line of sight, the rate of change in projected linear size increases approximately with the inverse square of viewing distance. The model’s projected boundaries may therefore diverge more rapidly from the keyframe AOIs within the analysis interval at 2 m, potentially increasing $N_{\mathrm{off}}$. Neither this motion-related AOI mismatch nor the depth-dependent mapping error was independently quantified; the observed reduction therefore cannot be attributed uniquely to either mechanism.

Across the three viewing distances, the upper-body AOI accounted for the largest percentage of the post-processing gaze samples ($N_{\mathrm{proc}}$), and the KDE maps showed that high-density gaze regions were generally concentrated around the predefined right-chest target. The upper-body AOI assignment percentage did not show a statistically detectable difference across viewing distances (p=0.105). Because participants were explicitly instructed to attend to the right-chest region, these distributions primarily reflect the system’s ability to localize instructed visual attention rather than spontaneous attentional preference. The consistency of the gaze-density maps with the predefined target nevertheless supports the feasibility of using the proposed framework for AOI-level attention measurement across changing observer-to-target distances.

### 4.2. Limitations and Future Work

First, Experiment 1 evaluated newly acquired post-calibration samples at the same spatial locations used for participant-specific calibration. The reported positional errors therefore characterize same-location post-calibration performance rather than held-out spatial generalization. Because the point-level calibration data required for reliable refitting were not retained, future work should evaluate independent spatial target sets or prespecified held-out calibration procedures. In addition, the Experiment 1 proposed-system metrics reflect the fixed operational preprocessing configuration. Intermediate POR outputs were not retained at every processing stage, precluding retrospective calculation of unfiltered or stage-specific precision measures, the exact Experiment 1 DBSCAN rejection rate, and systematic parameter-sensitivity analyses. The retained Experiment 1 outputs for the proposed system also did not include per-target mean gaze coordinates or sample-level POR coordinates, preventing retrospective calculation of the conventional target-referenced mean-position offset. This limits direct comparison of the proposed system with manufacturer specifications and studies that report accuracy using that definition. Future evaluations should retain sample-level outputs and report a metric-matched mean-position accuracy measure alongside $A_{\mathrm{spa}}$. Prospective studies should therefore retain stage-specific outputs and assess filtering and clustering sensitivity.

Second, Experiment 2 used a single planar calibration at 7 m for targets subsequently observed at 6, 4, and 2 m, and the resulting depth-dependent mapping mismatch was not independently quantified. In addition, the fixed DBSCAN radius (ε=40 px) and KDE bandwidth (h=30 px) represented different proportions of the projected body size across viewing distances. Consequently, cross-distance comparisons retain a processing-parameter component: DBSCAN may affect the composition of the processed sample set, whereas KDE affects only the visualized density distribution and does not enter AOI assignment or the statistical analyses. Experiment 2 also lacked independent frame-by-frame point-level gaze ground truth; the AOI-retained sample percentage should therefore be interpreted as a post-processing AOI-inclusion measure. Future work should evaluate multi-depth calibration and depth-aware scene mapping under controlled target-depth conditions.

Third, each experiment included 16 relatively young participants. Spectacle wearers and participants with myopia greater than 3.00D in magnitude were not evaluated. Future work should include larger and more diverse participant samples.

Fourth, the inherited eye-region landmark network was trained on synthetic UnityEyes imagery and was not fine-tuned or domain-adapted using the far-field RGB recordings collected in the present study. A domain mismatch therefore exists between the original training imagery and the present acquisition conditions, but its contribution to the observed end-to-end localization error was not independently quantified.

Fifth, Experiment 2 was conducted without mechanical head stabilization, but head position and orientation were not independently measured. Accordingly, the present study does not quantify head-motion tolerance or POR robustness as a function of head translation or rotation.

## 5. Conclusions

This study developed a far-field, non-contact eye-tracking framework for visual-attention measurement in fashion-runway settings. By integrating appearance-based 3D gaze-direction estimation, individualized scene mapping, and AOI-oriented attention analysis, the framework connects RGB-based gaze estimation with interpretable scene-level visual-attention measurement.

Experiment 1 characterized the operating performance of the integrated RGB-to-scene gaze-mapping pipeline under the controlled benchmark condition.

Although the proposed system showed greater point-level positional error and a higher processed-output $A_{\mathrm{spa}}$ value than the EyeLink 1000 Plus under the controlled benchmark, the proposed system maintained scene-level localization within a low-single-digit visual-angle range, supporting its intended use for broader AOI-level analysis. Experiment 2 further supported the feasibility of target-directed AOI localization across the tested viewing distances, although the AOI-retained sample percentage decreased at 2 m.

Overall, these findings support the feasibility of the proposed framework for far-field, non-contact AOI-level visual-attention measurement under the present simulated runway-observation conditions. Future work should evaluate the framework’s performance in unrestricted live-runway environments, including continuous smooth-pursuit and synchronized tracking of multiple observers.

## Figures and Tables

**Table 1:** Projected AOI extents across viewing distances.

Distance (m)	Head AOI	Upper-Body AOI	Lower-Body AOI
6	140×201 px	246×358 px	246×431 px
	(2.39°×3.44°)	(4.20°×6.11°)	(4.20°×7.34°)
4	210×302 px	369×537 px	369×646 px
	(3.58°×5.15°)	(6.30°×9.15°)	(6.30°×11.00°)
2	419×604 px	737×1074 px	737×1292 px
	(7.15°×10.29°)	(12.55°×18.18°)	(12.55°×21.79°)

Note. All dimensions are reported as width × height. Pixel extents represent the projected body-region dimensions in the 2560×1600 reference coordinate system; angular extents (in parentheses) were calculated from the corresponding physical body-region dimensions and viewing distances. The head AOI corresponds to the head-and-neck template.

**Table 2:** Results of Experiment 1.

Participant ID	EyeLink $A_{\mathrm{pos}}$, px (°)	EyeLink $A_{\mathrm{spa}}$, px (°)	Proposed $A_{\mathrm{pos}}$, px (°)	Proposed $A_{\mathrm{spa}}$, px (°)
1	44.37 (1.35°)	44.39 (1.35°)	83.04 (2.52°)	76.47 (2.32°)
2	55.75 (1.69°)	45.30 (1.38°)	83.08 (2.52°)	77.40 (2.35°)
3	60.65 (1.84°)	34.47 (1.05°)	71.30 (2.17°)	70.16 (2.13°)
4	46.80 (1.42°)	14.76 (0.45°)	82.78 (2.51°)	76.99 (2.34°)
5	34.79 (1.06°)	14.67 (0.45°)	92.92 (2.82°)	85.93 (2.61°)
6	61.51 (1.87°)	34.29 (1.04°)	79.64 (2.42°)	68.63 (2.08°)
7	60.94 (1.85°)	29.98 (0.91°)	88.10 (2.68°)	75.70 (2.30°)
8	66.82 (2.03°)	42.10 (1.28°)	80.86 (2.46°)	67.23 (2.04°)
9	114.18 (3.47°)	31.46 (0.96°)	101.96 (3.10°)	91.07 (2.77°)
10	45.11 (1.37°)	25.73 (0.78°)	84.01 (2.55°)	72.00 (2.19°)
11	57.93 (1.76°)	43.69 (1.33°)	86.34 (2.62°)	91.24 (2.77°)
12	62.28 (1.89°)	18.27 (0.55°)	75.85 (2.30°)	78.21 (2.38°)
13	42.70 (1.30°)	19.77 (0.60°)	65.03 (1.98°)	62.85 (1.91°)
14	39.23 (1.19°)	43.27 (1.31°)	82.60 (2.51°)	78.10 (2.37°)
15	57.84 (1.76°)	54.33 (1.65°)	77.43 (2.35°)	74.48 (2.26°)
16	63.93 (1.94°)	41.74 (1.27°)	80.08 (2.43°)	72.85 (2.21°)
Mean ± SD	57.18 ± 18.10 (1.74 ± 0.55°)	33.64 ± 12.21 (1.02 ± 0.37°)	82.19 ± 8.37 (2.50 ± 0.25°)	76.21 ± 7.90 (2.31 ± 0.24°)

Note. $A_{\mathrm{pos}}$ = combined target-referenced localization error, calculated as the mean sample-to-target Euclidean distance; $A_{\mathrm{spa}}$ = spatial precision operationalized as RMS spatial dispersion of the analyzed POR output. Pixel values are expressed in the common 1920×1080 coordinate frame, corresponding to 0.159cm/px. Visual-angle values were derived using the 3-m eye-to-canvas distance. Lower values indicate better performance.

**Table 3:** Experimental results under the 6 m condition.

Participant	$N_{\mathrm{proc}}$	Head AOI (% of $N_{\mathrm{proc}}$)	Upper-Body AOI (% of $N_{\mathrm{proc}}$)	Lower-Body AOI (% of $N_{\mathrm{proc}}$)	$N_{\mathrm{off}}$	AOI-Retained Samples (%)
1	256	45.7	52.0	0.0	6	97.7
2	219	13.2	71.7	0.0	33	84.9
3	205	1.0	98.0	0.0	2	99.0
4	237	0.0	94.9	0.0	12	94.9
5	191	15.2	77.5	0.0	14	92.7
6	200	16.0	81.5	0.0	5	97.5
7	273	19.4	76.2	0.0	12	95.6
8	220	6.4	93.6	0.0	0	100.0
9	222	0.0	96.8	0.0	7	96.8
10	219	26.5	62.1	0.0	25	88.6
11	212	21.7	74.1	0.0	9	95.8
12	229	9.2	88.6	0.0	5	97.8
13	200	11.0	87.0	0.0	4	98.0
14	192	4.7	93.2	0.0	4	97.9
15	225	0.0	100.0	0.0	0	100.0
16	224	2.7	95.5	0.0	4	98.2
Mean	–	12.0	83.9	0.0	–	95.96±4.10

**Table 4:** Experimental results under the 4 m condition.

Participant	$N_{\mathrm{proc}}$	Head AOI (% of $N_{\mathrm{proc}}$)	Upper-Body AOI (% of $N_{\mathrm{proc}}$)	Lower-Body AOI (% of $N_{\mathrm{proc}}$)	$N_{\mathrm{off}}$	AOI-Retained Samples (%)
1	202	9.9	87.1	0.0	6	97.0
2	276	17.4	82.6	0.0	0	100.0
3	294	16.7	71.4	0.0	35	88.1
4	184	41.3	56.0	0.0	5	97.3
5	208	38.0	62.0	0.0	0	100.0
6	188	35.6	63.3	0.0	2	98.9
7	141	7.1	84.4	0.0	12	91.5
8	226	34.1	60.6	0.0	12	94.7
9	251	24.7	71.7	0.0	9	96.4
10	242	17.4	82.6	0.0	0	100.0
11	246	35.4	64.6	0.0	0	100.0
12	250	31.2	63.2	0.8	12	95.2
13	162	28.4	71.6	0.0	0	100.0
14	265	39.2	60.8	0.0	0	100.0
15	274	52.2	45.6	0.0	6	97.8
16	209	57.4	42.6	0.0	0	100.0
Mean	–	30.4	66.9	0.05	–	97.31±3.50

**Table 5:** Experimental results under the 2 m condition.

Participant	$N_{\mathrm{proc}}$	Head AOI (% of $N_{\mathrm{proc}}$)	Upper-Body AOI (% of $N_{\mathrm{proc}}$)	Lower-Body AOI (% of $N_{\mathrm{proc}}$)	$N_{\mathrm{off}}$	AOI-Retained Samples (%)
1	182	0.0	61.0	0.0	71	61.0
2	226	0.0	89.8	0.0	23	89.8
3	206	0.0	100.0	0.0	0	100.0
4	201	0.0	98.0	0.0	4	98.0
5	203	0.0	71.9	0.0	57	71.9
6	295	0.0	54.6	0.0	134	54.6
7	228	0.0	60.1	3.1	84	63.2
8	251	0.0	70.5	0.0	74	70.5
9	271	0.0	80.1	0.0	54	80.1
10	289	0.0	77.5	5.5	49	83.0
11	248	0.0	89.9	0.0	25	89.9
12	236	0.0	75.0	10.6	34	85.6
13	228	0.0	60.5	0.0	90	60.5
14	246	38.6	48.4	0.0	32	87.0
15	187	14.4	84.5	0.0	2	98.9
16	184	0.0	92.9	0.0	13	92.9
Mean	–	3.3	75.9	1.2	–	80.43±14.95

## Data Availability

The original data presented in this study are openly available in Zenodo (version v2) at https://doi.org/10.5281/zenodo.22706802.
